# Timing the spinal cord development with neural progenitor cells losing their proliferative capacity: a theoretical analysis

**DOI:** 10.1186/s13064-019-0131-3

**Published:** 2019-03-13

**Authors:** Manon Azaïs, Eric Agius, Stéphane Blanco, Angie Molina, Fabienne Pituello, Jean-Marc Tregan, Anaïs Vallet, Jacques Gautrais

**Affiliations:** 1 Centre de Recherches sur la Cognition Animale (CRCA), Centre de Biologie Intégrative (CBI), Université de Toulouse; CNRS, UPS, Toulouse, France; 2 Centre de Biologie du Développement (CBD), Centre de Biologie Intégrative (CBI), Université de Toulouse; CNRS, UPS, Toulouse, France; 3 LaPlaCE, Université de Toulouse; CNRS, UPS, Toulouse, France

**Keywords:** CDC25B, Neural tube, Neural progenitors, Spinal cord, Proliferation, Differentiation, Proliferative capacity, Modeling

## Abstract

In the developing neural tube in chicken and mammals, neural stem cells proliferate and differentiate according to a stereotyped spatiotemporal pattern. Several actors have been identified in the control of this process, from tissue-scale morphogens patterning to intrinsic determinants in neural progenitor cells. In a previous study (Bonnet et al. eLife 7, 2018), we have shown that the CDC25B phosphatase promotes the transition from proliferation to differentiation by stimulating neurogenic divisions, suggesting that it acts as a maturating factor for neural progenitors. In this previous study, we set up a mathematical model linking fixed progenitor modes of division to the dynamics of progenitors and differentiated populations. Here, we extend this model over time to propose a complete dynamical picture of this process. We start from the standard paradigm that progenitors are homogeneous and can perform any type of divisions (proliferative division yielding two progenitors, asymmetric neurogenic divisions yielding one progenitor and one neuron, and terminal symmetric divisions yielding two neurons). We calibrate this model using data published by Saade et al. (Cell Reports 4, 2013) about mode of divisions and population dynamics of progenitors/neurons at different developmental stages. Next, we explore the scenarios in which the progenitor population is actually split into two different pools, one of which is composed of cells that have lost the capacity to perform proliferative divisions. The scenario in which asymmetric neurogenic division would induce such a loss of proliferative capacity appears very relevant.

**Electronic supplementary material:**

The online version of this article (10.1186/s13064-019-0131-3) contains supplementary material, which is available to authorized users.

## Introduction

How can a small number of apparently initially homogeneous neural stem cells (NSCs) give rise to the tremendous diversity of differentiated neurons and glia found in the adult central nervous system (CNS) ? The long-standing paradigm just claims: by proliferating first, and then restricting the kind of cells a progenitor can produce given its situation in time and space. How the progenitors fate progression occurs in different contexts is still under scrutiny.

In Drosophila, NSCs are multi-potent and divide asymmetrically to generate different types of progenies in a stereotypical manner. The study of mechanisms by which a single NSC can generate a wide repertoire of neural fates in this system is in fast progress [1]. In particular, several studies have highlighted the deterministic role of a series of sequentially expressed transcription factors in the temporal specification of Drosophila NSCs [2], albeit further studies substantiated that they are possibly under the control of some extrinsic (especially nutritional) factors [3].

In the mammalian cerebral cortex, the diversity of neural progenies has been linked to different types of cortical progenitors [4]. Beside expressing specific transcription factors, a set of criteria allows classifying the various types of cortical progenitors, including the apical or basal location of mitosis, their cell polarity and morphological features and proliferative capacity [5].

In the developing spinal cord, morphogen gradients have been identified that induce neural progenitor cells to express specific combinations of transcription factors and thereby adopt different identities based on their position along the dorsoventral axis [6–8]. This spatial patterning system ensures that different types of neurons are generated in an adequate stereotypical spatial order. The molecular players that control this spatial specification and their mode of action have been characterized [7]. However, little is known yet about how *temporal* differentiation of neural progenitor cells is orchestrated, namely what controls the timing of their transition from proliferation to differentiation at a given location [9].

Unlike cortical progenitors, spinal progenitors appear as a homogeneous population. They all divide apically and display the same morphology: an elongated shape with cytoplasmic connections to both the apical and basal surfaces. Spinal neural progenitors perform three modes of cell division: proliferative division that generates two progenitors (PP), asymmetric neurogenic division giving rise to a progenitor and a neuron (PN), and terminal neurogenic division producing two neurons (NN). The temporality of the transitions among the three modes of division (hereafter MoD) is critical in the control of the temporality of differentiation. Interestingly, we identified a G2/M cell cycle regulator, the CDC25B phosphatase whose expression correlates temporally and spatially remarkably well with areas where neurogenesis occurs [9, 10]. Moreover, CDC25B induces the conversion of proliferating neural progenitor cells into differentiating neurons by promoting sequentially neurogenic divisions, PN and NN [11]. We thus propose that CDC25B acts as a maturating factor that progressively restricts the mode of division of neural progenitor cells. Following our previous study on the maturing role of CDC25B in the control of neurogenesis [11], our question here is to examine whether this maturation can be due to an accumulating number of progenitors losing their proliferative capacity.

From that point of view, we note that ventral neural progenitors in the neural tube have been already shown to display a fate switch, transiting from early motoneurons production to late oligodendroglial production, under the control of Shh induction [12]. Here, we consider the possibility that a similar kind of switch operates sooner in the same population and sustains the transition from pure proliferative divisions to neurogenic divisions.

To examine this hypothesis, we start from the model of MoD transition we have proposed in our previous paper about the instrumental role that CDC25B plays in the progression from proliferative to neurogenic divisions [11]. In the spirit of Lander et al. [13], modeling is used here as a way to gain clarity in the face of intricacy. To this end, we have first extended our model presented in [11]. This model considered MoD as stationary over the 24 hours of our experiment. We now consider their change over time in order to extend this model over the full dynamics of ventral spinal cord motoneurons production. This extension over time uses the data published by Marty’s team [14] who measured the two essential components of this system at different times of spinal cord development: MoD on the one hand, and dynamics of Progenitors / Neurons (P/N) populations on the other hand.

From the modeling point of view, we point out the importance of being unequivocal about what the experimentally measured entities are in this system, and what are the conceptual entities we are thinking with. Namely, we propose below a first model which is based on the observable entities only (MoD and P/N evolutions). We use this model to make the link between these observable entities and check how experimentally measured evolution of modes of divisions can explain the evolution of cellular populations of progenitors (P-cells) and neurons (N-cells).

Next, we explore the idea that the temporality of the transitions among the three modes of division is based on a loss of proliferative capacity in some progenitors. To implement this hypothesis, we have to define two non observable kinds of progenitors, one of which is unable to perform proliferative divisions. We identify three scenarios compatible with this hypothesis. In order to check the structural consequences of each scenario, we reconstruct for each of them what the progression of their MoD should be if we take as a constraint that they must match the observable ones, and concurrently produce the correct evolution of P/N cells.

In the end, we advocate that one scenario is of great relevance: the hypothesis that asymmetric neurogenic division would induce the loss of proliferative capacity in the self-renewed progenitor. We offer a speculative additional component to the model so that robustness against small perturbations is secured. We discuss our findings compared to the model proposed by Marty’s team to explain their own data [14]. We finally suggest that lineage tracing may now be the best experimental avenue to go further in the understanding of how the progression from proliferative to neurogenic divisions is timed.

## Results

### Minimal Model for the Dynamics with three Modes of Division

We start from the model with fixed MoD proportions we designed in Bonnet et al. [11], incorporating here the possibility for the MoD to evolve with time. We consider a population of cells at time *t*, some of which are proliferating progenitors *P*(*t*), and others are differentiated neurons *N*(*t*). The dividing progenitors can undergo three kinds of division, yielding:

- symmetric proliferative divisions ending with two progenitors (pp-divisions)
- asymmetric self-renewing divisions ending with one progenitor and one neuron (pn-divisions)
- symmetric consumptive neurogenic divisions ending with two neurons (nn-divisions)

Let us denote : *η* the rate at which P-cells undergo divisions (in fraction of the P-pool per unit time) *α*_*pp*_(*t*) the fraction of dividing cells undergoing pp-divisions *α*_*pn*_(*t*) the fraction of dividing cells undergoing pn-divisions *α*_*nn*_(*t*) the fraction of dividing cells undergoing nn-divisions

The fractions of pp-, pn- and nn-divisions can evolve with time, under the constraint that *α*_*pp*_(*t*)+*α*_*pn*_(*t*)+*α*_*nn*_(*t*)=1.

The time derivative $\dot {P}(t)$ of pool *P*(*t*) (resp. $\dot {N}(t)$) is then given by the balance equation at time *t*, reading:

1 $$ \left\{ \begin{aligned} \dot{P}(t) &= -\eta(t) P(t) &+2\alpha_{pp}(t)\eta(t) P(t) +1\alpha_{pn}(t)\eta(t) P(t)\\ \dot{N}(t) &= &+ 2\alpha_{nn}(t)\eta(t) P(t) + 1\alpha_{pn}(t)\eta(t) P(t) \end{aligned} \right.  $$

where in the first equation :

−*η*(*t*)*P*(*t*) quantifies the rate at which P-cells disappear from the pool *P*(*t*) because they divide. The quantity of disappearing P-cells between *t* and *t*+*dt* is then *η*(*t*)*P*(*t*)*dt*
- *α*_*pp*_(*t*)*η*(*t*)*P*(*t*) quantifies the fraction of this quantity that undergoes a pp-division ; it doubles to yield 2 P and adds up to the pool P(t) (hence the factor 2)
- *α*_*pn*_(*t*)*η*(*t*)*P*(*t*) quantifies the fraction of this quantity that undergoes a pn-division ; it doubles to yield 1 P and 1 N, so only half (the P part) adds up to the pool P(t) (hence the factor 1)

Correspondingly in the second equation :

- *α*_*nn*_(*t*)*η*(*t*)*P*(*t*) quantifies the fraction of this quantity that undergoes a nn-division ; it doubles to yield 2 N and adds up to the pool N(t) (hence the factor 2)
- *α*_*pn*_(*t*)*η*(*t*)*P*(*t*) is the fraction of this quantity that undergoes a pn-division ; it doubles to yield 1 P and 1 N and only half (the N part) adds up to the pool N(t) (hence the factor 1)

System (1) is a textbook continuous-time representation of population dynamics. It is a very good approximation of the evolution of progenitors and neurons, considering that division events are instantaneous (M-phase is very short compared to the cell cycle duration), and occur uniformly in time (asynchronously) [11].

Since *α*_*pp*_+*α*_*pn*_+*α*_*nn*_=1, system (1) can be rewritten:

2 $$ \left\{ \begin{array}{ll} \dot{P}(t) &= (\alpha_{pp}(t) - \alpha_{nn}(t)) \eta P(t)\\ \dot{N}(t) &= \left(1-(\alpha_{pp}(t) - \alpha_{nn}(t))\right) \eta P(t) \end{array} \right.  $$

so that the general form of the solution for the evolution of the pools is given by:

3 $$ \left\{ \begin{aligned} P(t) &= P(0)\exp\left[{\int_{0}^{t}(\alpha_{pp}(\tau) - \alpha_{nn}(\tau))\eta(\tau) d\tau}\right] \\ N(t) &= N(0) + \int_{0}^{t} \left(1-(\alpha_{pp}(\tau) - \alpha_{nn}(\tau))\right) \eta(\tau) P(\tau) d\tau \end{aligned} \right.  $$

Starting from an initial configuration, *P*(0)=1,*N*(0)=0 at time *t*_0_ and considering a steady rate *η*(*t*)=*η*, the system evolution will be only driven by the two functions *α*_*pp*_(*t*) and *α*_*nn*_(*t*).

### Calibration from data for the embryonic spinal cord

In the embryonic spinal cord, pp-divisions are largely dominant at the beginning of the process so that proliferation increases the pool of progenitors for a while, but their proportion decreases with time so that the process ends with terminal neurogenic divisions.

**Fig. 1:**
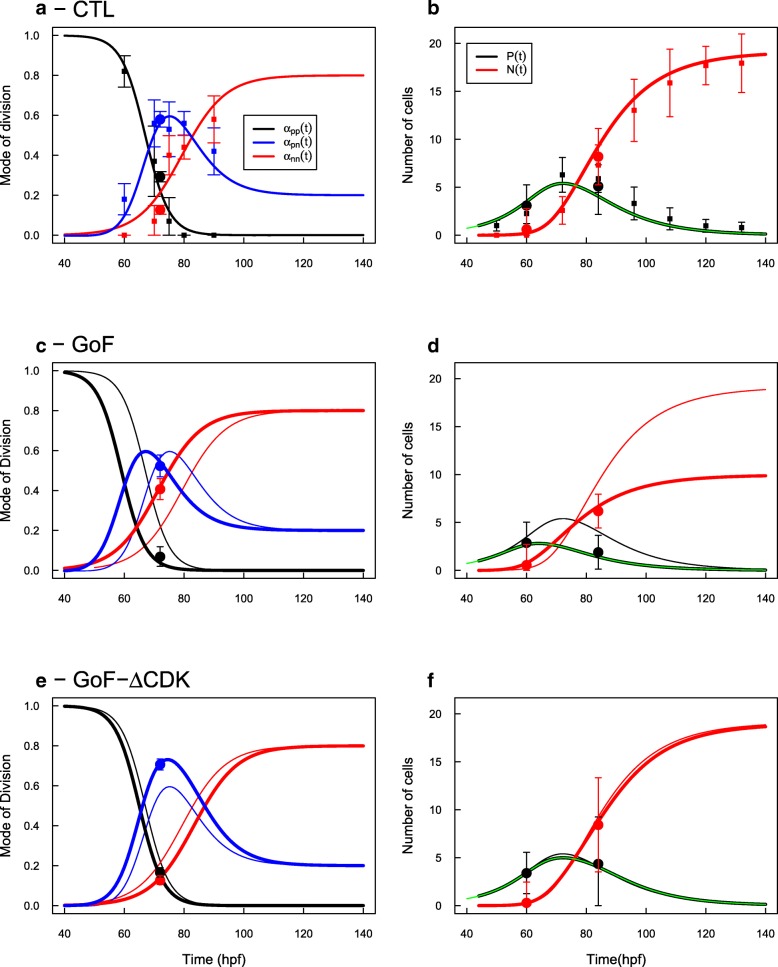
PN Model for the dynamics of Modes of Division (MoD) and evolution of cells population (P,N) in the developing ventral spinal cord. **a** MoD measured by [14] (square dots) and [11] (circles, bars are 95% CI). Black : pp-divisions, red : nn-divisions, blue : pn-divisions. Curves report the fitted continuous time functions. **b** Evolutions of the pools of progenitors (black) and neurons (red) from [14]. Circle points indicate estimates of P/N proportion from [11], and scaled to the total amount of cells. Black and red lines report numerical solution of system (3) using MoD shown in a). Green line reports the analytical solution for the P-pool (Eq. 6). **c** CDC25B Gain-of-Function promotes neurogenic divisions so that the transition from proliferation to differentiation is shifted 8 hours sooner (thick lines) than the CTL profiles (thin lines). **d** Predicted evolution of the pools of progenitors (black) and neurons (red) under GoF (thick lines) compared to CTL (thin lines). The dots report the proportion of progenitors / neurons measured in Bonnet et al. in GoF condition [11], scaled to the total amount predicted at their respective times. **e** CDC25B- *Δ*CDK Gain-of-Function have a differential effect upon neurogenic divisions: pp-divisions are shifted 2 hours sooner and nn-divisions are shifted 4 hours later. As a consequence, the complementary PN profile is enhanced (compared to the CTL) and lasts longer. **f** The dynamics of the two pools is very close to the CTL dynamics and match with the measured proportions given in Bonnet et al. [11]

Estimations of MoD were collected by Saade et al. [14] at discrete times (Fig. 1a), as well as the corresponding evolutions of the pools of progenitors and neurons (Fig. 1b). We used these MoD data to calibrate the two continuous time functions *α*_*pp*_(*t*) and *α*_*nn*_(*t*), with *α*_*pn*_(*t*) being constrained to be their complement to 1 (Fig. 1a, Additional file 1).

From a minimalistic approach, we constrain the shape of the two functions with a minimal set of parameters. The pp-divisions display an evolution from *α*_*pp*_(*t*_0_)=1 down to *α*_*pp*_(*t*→*∞*)=0. This transition will be characterized by a characteristic time *τ*_*pp*_, with *α*_*pp*_(*τ*_*pp*_)=0.5, and a characteristic scale *σ*_*pp*_ indicating the sharpness of transition. A standard form for this is:

4 $$ \alpha_{pp} (t) = \frac{1}{2} \left[1 - \text{tanh} \left(\frac{t - \tau_{pp}}{\sigma_{pp}} \right)\right]  $$

Least-square error estimation of the two parameters yields: *τ*_*pp*_=67.0 hpf and *σ*_*pp*_=8.0 hpf. The adjusted profile fits the data rather well (sq. error = 0.007).

We fit the same kind of tanh profile for the evolution of nn-divisions from *α*_*nn*_(*t*_0_)=0 to *α*_*nn*_(*t*→*∞*), following:

5 $$ \alpha_{nn} (t) = \frac{1}{2} \alpha_{nn}(t \rightarrow \infty) \left[1 + \text{tanh} \left(\frac{t - \tau_{nn}}{\sigma_{nn}} \right)\right]  $$

We lack the data to fit exactly the plateau value and we set the reasonable value *α*_*nn*_(*t*→*∞*)=0.8. Least-square error estimation of the two parameters yields: *τ*_*nn*_=79.3 hpf and *σ*_*nn*_=14.5 hpf (sq. error = 0.03).

With these profiles for *α*_*pp*_(*t*) and *α*_*nn*_(*t*), the evolution of the P-pool evolves according to (details in Methods Eq. 18):

6 $$ {\begin{aligned} \frac{P(t)}{P(0)} =\exp\left[ \frac{\eta}{2} \right.& \left(\left[ t - \sigma_{pp}\ln \left(\frac{\cosh((t-\tau_{pp})/\sigma_{pp})}{\cosh(-\tau_{pp}/\sigma_{pp})}\right) \right]\right.\\ &\left.\left.- \alpha_{nn,\infty} \left[ t + \sigma_{nn}\ln \left(\frac{\cosh((t-\tau_{nn})/\sigma_{nn})}{\cosh(-\tau_{nn}/\sigma_{nn})}\right) \right] \right) \right] \end{aligned}}  $$

Setting *P*(0)=1,*N*(0)=0 at time *t*_0_=44 hpf and *η*=1/12 hours [11, 15], this system yields a good account of the evolution of P,N pools as measured by Saade et al. [14] (Fig. 1b, original data were rescaled to correspond to the number of cells per progenitor originally present). At the beginning, the large bias towards pp-divisions amplifies the pool of progenitors up to a maximum value: *P*_*max*_[*CTL*]=5 per initial progenitor at around *t*_*maxp*_[*CTL*]=72 hpf. Then, the production of neurons raises mainly due to pn-divisions, until nn-divisions become dominant over pn-divisions (at around 82-83 hpf). The pool of progenitors depletes to zero while nn-divisions increase the pool of neurons up to a plateau value of *N*(*t*→*∞*)[*CTL*]=17.6 neurons per progenitor initially present. We note that this evolution, and especially *N*(*t*→*∞*) is highly sensitive to the chosen initial condition (*t*_0_,*P*(*t*_0_)). This point is addressed below.

#### Incorporating CDC25B experiments

Bonnet et al. [11] have performed a series of experimental manipulations of the expression of CDC25B phosphatase in this biological system. Their experimental measures are the proportions of progenitors / neurons, and a corresponding measure of the modes of division, depending on the experimental conditions : Control (CTL), Gain of Function (CDC25B GoF) using the wild-type form of CDC25B, and Gain of Function using a CDC25B modified to be unable to interact with its known substrates CDKs (CDC25B ^*Δ*CDK^ GoF).

Modes of division were measured by Bonnet et al. [11] at stage HH17, and fit well with the MoDs measured by Saade et al. [14] at time 72 hpf (Fig. 1-a, circle dots). However, to make the correspondence between P/N fractions reported in Bonnet et al. [11] and the P/N evolution measured in Saade et al. [14], we had to consider that the former correspond respectively to times 60 hpf and 84 hpf on the time scale in Saade et al. [14] (i.e. 12 h before and after 72 hpf, keeping the correct interval of 24h in between).

To check the power of this simple model, we now explore the hypothesis that CDC25B GoF has only an effect upon the schedule of MoD transitions. We expect that GoF should trigger differentiation sooner in time, and indeed, the measured MoD in the GoF experiment can be fitted by shifting the three time profiles 8 hours sooner (Fig. 1-c). Interestingly, at time 72 hpf, this strongly affects *α*_*pp*_ and *α*_*nn*_ but leaves *α*_*pn*_ unchanged.

The corresponding evolutions of the pools P/N are strongly affected, since the progenitors lack time to proliferate, reaching now a maximum of *P*_*max*_[*GoF*]=2.6 per initial progenitor at around *t*_*maxp*_[*GoF*]=64 hpf (Fig. 1-d). As a consequence, the pool of neurons increases sooner, but reaches a plateau value nearly half of that of the CTL condition, *N*(*t*→*∞*)[*GoF*]=9.2 neurons per initial progenitor. The proportions P/N measured by Bonnet et al. [11] fit well with this picture.

The case of CDC25B ^*Δ*CDK^ GoF yields a different prediction. Here, the pp-divisions had to be advanced by 2 hours while the nn-divisions had to be delayed by 4 hours to correspond to the ones measured by Bonnet et al. [11] (Fig. 1e). As a result, the main effect of CDC25B ^*Δ*CDK^ GoF is to greatly promote pn-divisions, so they appear sooner and reach a higher proportion. This suggests that CDC25B ^*Δ*CDK^ GoF promotes self-renewing neurogenic pn-divisions, but fails to promote the transition from pn-divisions to nn-divisions as does CDC25B GoF.

Here again, the predicted dynamics of the two pools fit well the proportions P/N measured by Bonnet et al. [11] (Fig. 1f). Remarkably, since the pn-divisions are neutral to the balance proliferation / differentiation, these dynamics are almost identical to the CTL case. We note that the effect of CDC25B- *Δ*CDK could not be detected by measuring only the P/N pools evolution.

Altogether, the model given by the system 1 (PN model) expresses the dynamics at the population scale, yielding the evolution of the two kinds of cells: the pool of progenitors, and the pool of neurons. Being formulated at the population scale, the variables and the parameters represent averages over a large ensemble of cells. In the biological system, those averages can correspond to numerous scenarios at the cell level. Nonetheless, the model dynamics produced by Eqs. 3, 4, 5 should be taken as a point of reference because any scenario at the cell scale should reproduce these dynamics at the population scale. In that sense, PN model should be regarded as a way to describe a strong constraint over the set of possible cell-scale scenarios and a guide to narrow the research of mechanistic explanations. In the next section, we will use it as such in order to explore three scenarios incorporating a loss of proliferative capacity at the cell scale as a means to time the progression from proliferative to purely neurogenic divisions.

### Models with loss of proliferative capacity

PN model is compatible with the simplest interpretation at the cell level: that each dividing cell is liable to stochastically produce the three possible MoD, in proportion to what is measured at the population scale. Since the data show that progenitors MoD display an irreversible vanishing of pp-divisions with time, we now explore alternative models in which we explicitly introduce loss of proliferative capacity at the cell scale, so that more and more dividing progenitors cannot perform proliferative divisions.

This loss of proliferative capacity at the cell scale implies that the pool of progenitors is actually composed of different kinds of dividing cells. Let’s consider the case with only two kinds of dividing cells, denoted *G* and *A*, where only cells of type G are able to perform proliferative divisions (*G*→(*G*,*G*)). *A*-cells would be produced by non proliferative MoD of *G*-cells when they stochastically adopt the alternative MoD, producing daughter cells with no proliferative capacity. The total pool of dividing cells (progenitors in the model 1) becomes *P*(*t*)=*G*(*t*)+*A*(*t*).

The loss of proliferative capacity in cells of type *A* implies that they cannot give birth to a cell of type *G* nor perform proliferative divisions (*A*→(*A*,*A*)). Hence, they can only undergo asymmetric self-renewing neurogenic division *A*→(*A*,*N*) or symmetric consumptive neurogenic division *A*→(*N*,*N*).

The only choice left then is to define the pair of cells produced by non proliferative MoD of *G*-cells. The only four possibilities are:

1. *G*→(*G*,*A*) : asymmetric non-neurogenic division
  One cell keeps proliferative capacity (keeps type G) and one cell loses it (becomes type A).
2. *G*→(*A*,*A*): symmetric non-neurogenic division
  The two daughter cells lose proliferative capacity but keep self-renewing capacity (both become type A).
3. *G*→(*A*,*N*): asymmetric neurogenic division
  Both cells lose proliferative capacity, with one cell keeping self-renewing capacity (becomes type A) and the other cell will become a neuron.
4. *G*→(*N*,*N*): symmetric neurogenic division
  The two cells will become neurons, with no proliferative nor self-renewing capacity.

Using the nomenclature established in [5], the types and effects of those MoD are summarized in Table 1.

**Table 1:** Description of the MoD in the three models with loss of proliferative capacity

MoD	Type	Effect	Present in model
*G*→(*G*,*G*)	Symmetric proliferative	Proliferative	GGA, GAA, GAN
*G*→(*G*,*A*)	Asymmetric self-renewing	Proliferative	GGA
*G*→(*A*,*A*)	Symmetric consumptive	Proliferative	GAA
*G*→(*A*,*N*)	Asymmetric consumptive	Neurogenic	GAN
*G*→(*N*,*N*)	Symmetric consumptive	Neurogenic	PN
*A*→(*A*,*N*)	Asymmetric self-renewing	Neurogenic	GGA, GAA, GAN
*A*→(*N*,*N*)	Symmetric consumptive	Neurogenic	GGA, GAA, GAN

In symmetric divisions, the two daughter cells display the same identity. In asymmetric divisions, the two daughter cells have different identities. In self-renewing divisions, one of the daughter cells has the same identity as the mother cell. In consumptive divisions, the two daughter cells differ in identity from the mother cell. In neurogenic divisions, at least one daughter cell is a neuron

We note that the fourth possibility would correspond to PN model (since no cell of type A would even be produced), but with such parameters that no asymmetric division would appear at all. We discard it in the spinal cord context since asymmetric divisions are observed. We examine below the three other scenarios, naming them after the specific non-proliferative MoD of the G-cells: GGA-model, GAA-model and GAN-model.

#### Structural flaw of GGA model

Under the GGA model, the MoD are : *G*→(*G*,*G*) and *G*→(*G*,*A*) for the G-cells. They can then perform either proliferative divisions or self-renewing divisions. As a consequence, this model cannot structurally account for the decreasing of the P-pool after 73 hpf. Even if their MoDs evolve from proliferative in the beginning to self-renewing in the end, the early proliferation would lead to a given amount of G-cells that could not decrease later and the G-pool would stabilize. When stabilized, it would continuously produce A-cells at a constant rate by self-renewing division. Since these A-cells would in turn differentiate into neurons, that would produce a population of neurons growing to infinite: the structure of the model would trap the dynamics in a perpetual regime of permanent production of neurons. This model is then to be rejected because of its structure. Incidentally, we note that this rejection based on the structure of the model is an indication that not *any* model with loss of proliferative capacity could fit the observed dynamics.

#### Predictions of GAN model

Writing explicitly the balance of evolution, the dynamics of GAN model obeys:

7 $$ \left\{ \begin{aligned} \dot{G}(t) & = \eta \, \left[ -G(t) + 2 \alpha_{GGG}(t)G(t) \right]\\ \\ \dot{A}(t) & = \eta \, \left[-A(t) + \alpha_{GAN}(t)G(t) + \alpha_{AAN}(t)A(t) \right]\\ \\ \dot{N}(t) & = \eta \, \left[ \alpha_{GAN}(t)G(t) + 2 \alpha_{ANN}(t)A(t) \,+\, \alpha_{AAN}(t)A(t) \right] \\ \\ & \alpha_{GGG}(t) + \alpha_{GAN}(t)= 1 \; ; \; \alpha_{AAN}(t)+ \alpha_{ANN}(t) = 1 \end{aligned} \right.   $$

Let’s denote *γ*_*G*_(*t*)=*α*_*GAN*_(*t*) and *γ*_*A*_(*t*)=*α*_*ANN*_(*t*).

Using the fourth line of system (7), system (7) simplifies to (omitting time dependencies for clarity):

8 $$ \left\{ \begin{aligned} \dot{G} & = \eta \, \left(1-2\gamma_{G}\right) G\\ \dot{A} & = \eta \, \left(\gamma_{G}G -\gamma_{A}A \right)\\ \dot{N} & = \eta \, \left(\gamma_{G}G + (1+\gamma_{A})A \right) \\ \end{aligned} \right.   $$

showing that the evolution is fully determined by *γ*_*G*_(*t*) and *γ*_*A*_(*t*). To calibrate these two time-continuous functions, we will use the evolutions of MoD in the PN model for the three conditions (CTL, CDC25B GoF, CDC25B ^*Δ*CDK^ GoF).

For this, we establish the correspondence between GAN model variables and PN model variables :

9 $$ \left\{ \begin{aligned} P(t) &= G(t) + A(t) \\ \alpha_{pp}(t) &= (1-\gamma_{G}(t))\frac{G(t)}{G(t) + A(t)}\\ \\ \alpha_{pn}(t) &= \gamma_{G}(t)\frac{G(t)}{G(t) + A(t)} + (1-\gamma_{A}(t))\frac{A(t)}{G(t) + A(t)}\\ \\ \alpha_{nn}(t) &= \gamma_{A}(t) \, \frac{A(t)}{G(t) + A(t)} \\ \\ \end{aligned} \right.  $$

To establish this correspondence, we have considered that the observable *α*_∙∙_(*t*) functions express the proportions of each MoD among a total number of divisions. They can be regarded as a probability that a given division is of a given kind of MoD. Hence, to reconstruct a given observable MoD, we have to multiply the probability that the corresponding kind of progenitor would adopt this MoD by the proportion of this kind of progenitors among the total number of progenitors. For instance, the probability observing a pp-division, *α*_*pp*_(*t*) (the observable proportion of proliferative divisions), is the probability that a given progenitor is of type *G* (namely *G*(*t*)/(*G*(*t*)+*A*(*t*))) times the probability that this progenitor performs an *G*→(*G*,*G*) division (*α*_*GGG*_(*t*)=1−*γ*_*G*_(*t*)). We proceed this way for the three kinds of observable MoD, considering that the observed asymmetric divisions *α*_*pn*_ aggregate the asymmetric divisions *G*→(*A*,*N*) by the *G* pool and asymmetric divisions *A*→(*A*,*N*) by the *A* pool.

As shown in Methods, analytical inversion of the evolution of *γ*_*G*_ can be matched very well by a tanh ansatz, so we used the same function for the evolution of *γ*_*A*_. To calibrate *γ*_*G*_(*t*) and *γ*_*A*_(*t*), we used the continuous time functions fitting the MoD in PN model to fit the two parameters of this ansatz by a least-square error procedure (full details are given in Methods “GAN calibration”).

**Fig. 2:**
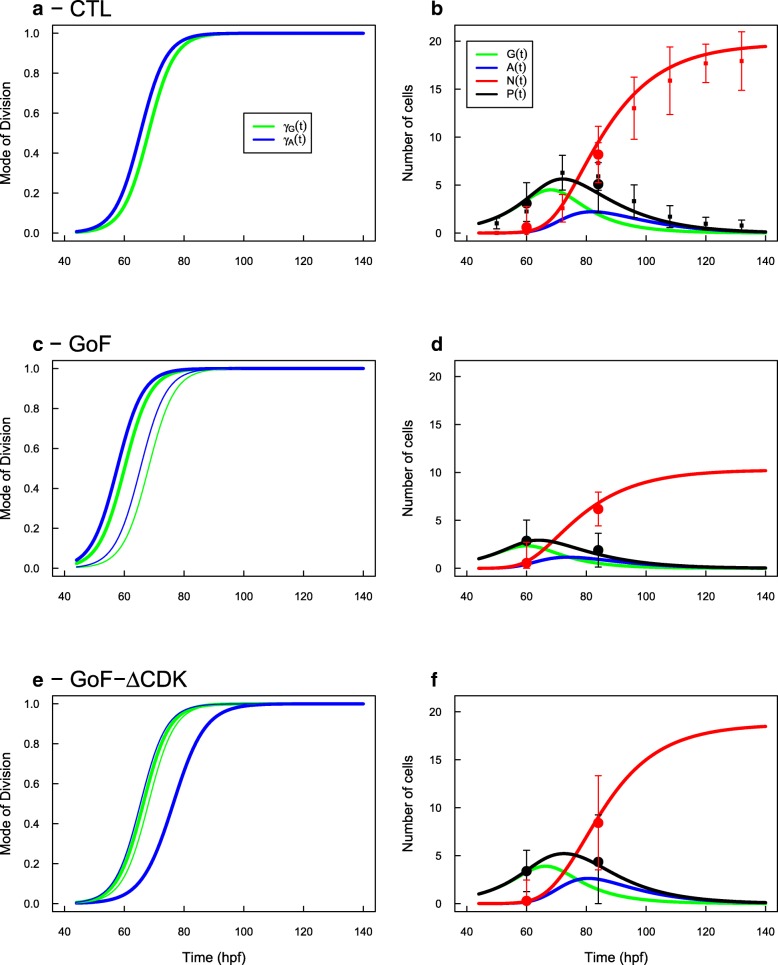
GAN Model. The fitted evolutions of MoD of G-cells (*γ*_*G*_) and A-cells (*γ*_*A*_) (left column) and their respective predictions for the evolutions of populations (right column) are reported for the three experimental conditions. In the two GoF conditions, the thin lines report the CTL condition for eye-comparison. Under the CTL condition, the evolutions of the two MoD are very similar (**a**). Under GoF of the wild-type CDC25B, both evolutions are shifted sooner in time by the same delay (8 h, **c**). Under GoF of the mutated form of CDC25B, only the evolution of A-cells MoD is affected, being delayed by 11 hours (**e**). In the three cases, the fitted MoD predict evolutions of progenitors (P=G+A) and neurons (N) in accordance with the data (**b**, **d**, **f**)

The fitted parameters are reported in Table 2, and the corresponding predictions for the evolutions of cells population are given in Fig. 2.

**Table 2:** Parameters found for the GAN model (in hpf)

	CTL	CDC25B GoF	CDC25B ^*Δ*CDK^ GoF
*τ*_*G*_	68.1	60.1	66.3
*τ*_*A*_	65.4	57.6	76.4
*σ*_*G*_	8.7	8.7	8.7
*σ*_*A*_	8.6	8.6	10.7

In the CTL case, we found a remarkable convergence of the MoD evolutions for *G*-cells and *A*-cells and we recover a perfect prediction for the evolution of *P*(*t*) and *N*(*t*) populations.

The typical time of MoD progression is 68hpf for the *G*-cells and 65.5hpf for the *A*-cells, and their progression rates are practically identical. In the beginning, the *G*-pool is mainly proliferating, while *G*→(*G*,*G*) is dominant over *G*→(*A*,*N*), for about 20 hours (Fig. 2a, green, *γ*_*G*_(*t*)<0.5 before 68 hpf). This yields a growth of the *G* pool up to a peak at 4.5 *G*-cells (per initial *G*-cells) at 68 hpf (Fig. 2b, green). They represent 88% of *P*-cells at that time. After that peak, *G*-cells slowly decreases while populating *A* and *N* cells through *G*→(*A*,*N*) divisions. From that time, *A*-cells are produced up to a peak from which terminal neurogenic divisions *A*→(*N*,*N*) become dominant so the *A*-pool decreases and neural production ends, with about 20 neurons per initial progenitor.

We note that the MoD of *A*-cells are already very skewed in favor of *A*→(*N*,*N*) at the time they begin to be produced by *G*→(*A*,*N*) divisions (Fig. 2a, blue, *γ*_*A*_(*t*)>0.65 after 68 hpf). Hence, most *A*-cells are consumed by terminal divisions as soon as they are produced. Seeing this, we checked an even simpler scenario with only three modes of division: *G*→(*G*,*G*), *G*→(*A*,*N*), *A*→(*N*,*N*) so a progenitor issued from an asymmetric division (A-cells) would always differentiate into two neurons at the next cycle. This yields practically the same results (Additional file 2: Figure S1).

In the CDC25B GoF case, the 8-hours advanced evolution of the MoD in PN model directly translates into an equivalent and parallel 8-hours advanced evolution for *G* and *A* MoD, which is not surprising given the calibration method.

Contrastingly, the evolution of these MoD differs in the case of CDC25B ^*Δ*CDK^ GoF. As expected, the small advanced *α*_*pp*_ profile little affects the progression of *G*-cells MoD. However, the 4-hours delayed *α*_*nn*_ profile translates into a threefold larger delay for the *A*-cells MoD, namely they are shifted 11-hours later than in the CTL condition (76.4 hpf vs 65.4 hpf). As a consequence, the *A*→(*A*,*N*) MoD becomes operative since it is still around 0.5 when *A*-cells reach their peak. In the end, the production of neurons is very similar to the CTL value.

Overall, this structure for introducing a type of cells with no more proliferative capacity appears perfectly compatible with the available data. Under this model, the evolutions of the MoD have two striking features: they show a monotone progression, and they are very similar to each other, opening the possibility that they could be under the control of a same regulation process (see below).

#### Predictions of GAA model

The dynamics of this model obey:

10 $$ \left\{ \begin{aligned} \dot{G}(t) &= \eta \, \left[ -G(t) + 2 \alpha_{GGG}(t) G(t) \right]\\ \\ \dot{A}(t) &= \eta \, \left[ -A(t) + 2 \alpha_{GAA}(t) G(t) + \alpha_{AAN}(t)A(t) \right]\\ \\ \dot{N}(t) &= \eta \, \left[ 2 \alpha_{ANN}(t)A(t) + \alpha_{AAN}(t)A(t) \right]\\ \\ &\alpha_{GGG}(t) + \alpha_{GAA}(t)= 1 \; ; \; \alpha_{AAN}(t)+ \alpha_{ANN}(t) = 1 \end{aligned} \right.  $$

Denoting *γ*_*G*_(*t*)=*α*_*GAA*_(*t*) and *γ*_*A*_(*t*)=*α*_*ANN*_(*t*), system (10) simplifies to:

11 $$ \left\{ \begin{aligned} \dot{G} & = \eta \, \left(1-2\gamma_{G}\right) G\\ \dot{A} & = \eta \, \left(2\gamma_{G}G -\gamma_{A}A \right)\\ \dot{N} & = \eta \, (1+\gamma_{A})A \\ \end{aligned} \right.   $$

The correspondences between GAA scenario variables and the variables in PN model are:

12 $$ \left\{ \begin{aligned} \alpha_{pp}(t) &= (1-\gamma_{G}(t)) \, \frac{G(t)}{G(t) + A(t)} + \gamma_{G}(t)\, \frac{G(t)}{G(t) + A(t)} \\ \\ \alpha_{pn}(t) &= (1-\gamma_{A}(t)) \, \frac{A(t)}{G(t)+A(t)} \\ \\ \alpha_{nn}(t) &= \gamma_{A}(t) \, \frac{A(t)}{G(t) + A(t)} \\ \\ P(t) &= G(t) + A(t) \\ \end{aligned} \right.  $$

**Fig. 3:**
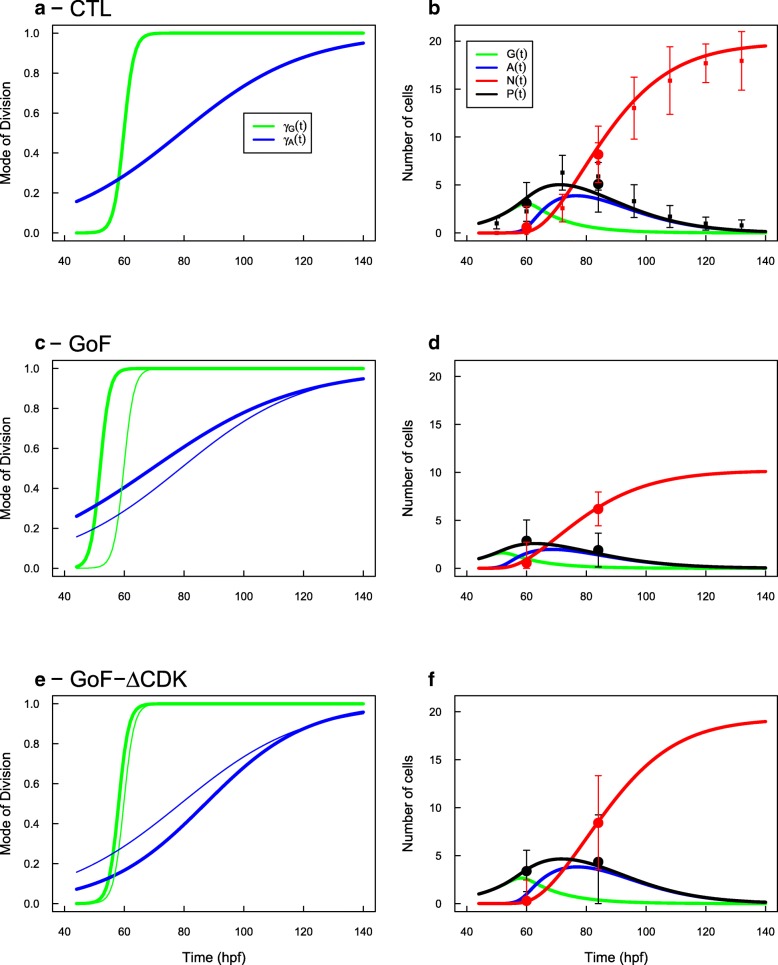
GAA Model. Same conventions as Fig. 2: evolutions of MoD under the three experimental conditions (**a**, **c**, **e**) and corresponding predictions for progenitors and neurons (**b**, **d**, **f**). Under the GAA model, the evolutions of the two MoD are very different in the CTL condition : G-cells switch to *G*→ (*A*,*A*) MoD early in the process while A-cells keep dividing by self-renewing division *A*→(*A*,*N*) for a long time to compensate the lack of proliferation. Under GoF, both transitions are shifted sooner in time (by 8 hours). Under GoF of mutated CDC25B, only the evolution of A-cells MoD is affected, being delayed by 8 hours. In the three cases, the fitted MoD predict evolutions of progenitors (P=G+A) and neurons (N) in accordance with the data

We used MoD fitted in PN model to calibrate the two MoD functions *γ*_*G*_(*t*) and *γ*_*A*_(*t*) the same way as we did for GAN model (full details in Methods “GAA calibration” section). The fitted parameters are given in Table 3 and the predicted evolutions are given in Fig. 3.

**Table 3:** Parameters found for the GAA model (in hpf)

	CTL	CDC25B GoF	CDC25B ^*Δ*CDK^ GoF
*τ*_*G*_	59.8	51.9	58.1
*τ*_*A*_	78.9	69.4	87.2
*σ*_*G*_	3.4	3.2	3.4
*σ*_*A*_	41.5	48.6	33.8

Under CTL condition, we observe an abrupt and early switch of the *G*-cells MoD, from dominant *G*→(*G*,*G*) MoD before 60 hpf to dominant *G*→(*A*,*A*) MoD after 60 hpf (Fig. 3a, green). As a consequence, the P-pool is made of only *G*-cells up to that time (Fig. 3b, black and green curves). After that proliferative burst, G-cells mainly differentiate into *A*-cells, and the former become dominant in the system (Fig. 3b, blue curve). Contrastingly, the MoD of *A*-cells evolves smoothly (Fig. 3a, blue) and the characteristic time of their switch is as late as 79 hpf. This leaves time for *A*-cells to produce neurons by self-renewing divisions *A*→(*A*,*N*) and to compensate for the early stopping of proliferative divisions by G-cells. After 79 hpf, *A*-cells engage more and more in terminal differentiation until their extinction.

The evolutions of *P*=*G*+*A* and *N* pools produced by these calibrated MoD match very well the measured ones (Fig. 3b, black and red curves).

In CDC25B GoF condition, the 8-hours advance of MoD in PN model is directly reflected in the MoD for the *G*-cells (Fig. 3c). This is expected given the calibration procedure, and this is true also for the progression of the MoD for the *A*-cell, although their slopes are further smoothened. This results into P/N evolutions under GoF condition that match the profiles under PN model (Fig. 3d).

In CDC25B ^*Δ*CDK^ GoF condition, the switch of MoD for the *G*-cells happens slightly sooner than in CTL condition (Fig. 3d, green), so that the total number of *A* cells produced by *G*→(*A*,*A*) is a bit lower (and hence so are *P*=*G*+*A* cells). On the contrary, the switch of MoD for the *A*-cells are delayed by about 5 hours (Fig. 3e, blue). This is consistent with the observation that pn-divisions in PN model are favored under CDC25B ^*Δ*CDK^ GoF condition where they operate for a longer time than in the CTL condition. Eventually, *A*-cells are fewer but self-renew longer and yield the same number of neurons as in CTL condition in the end.

Overall, this structure for introducing a type of cells with no more proliferative capacity also appears compatible with the available data for the P/N evolutions. We note however that the MoD profiles obtained by analytical inversion do not fit the MoD fitted to the ansatz (details in Methods “GAA calibration” section).

### Models comparison

**Fig. 4:**
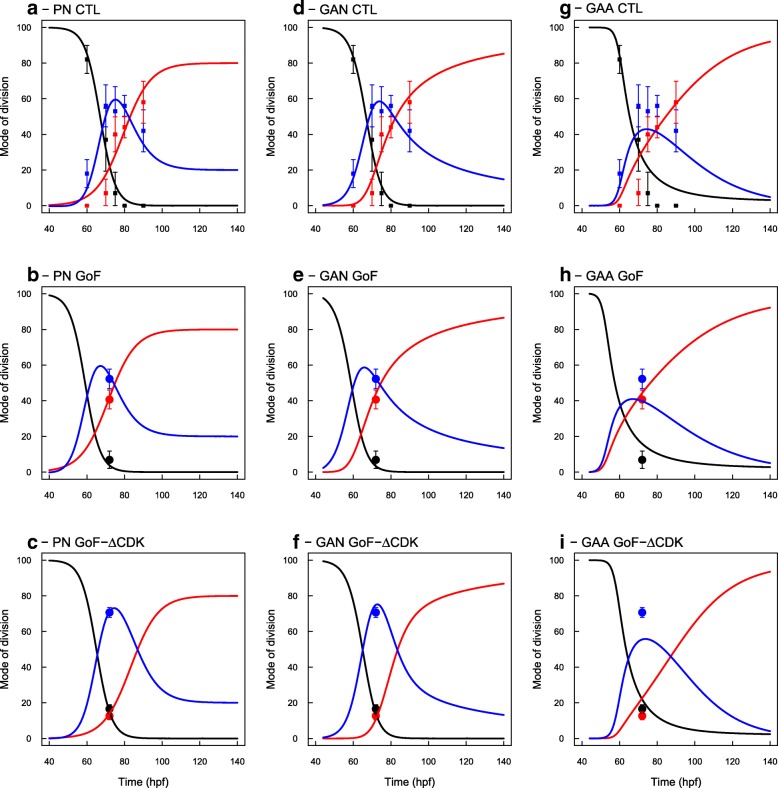
Compatibility of models PN, GAN and GAA regarding the MoDs. The fitted MoD in the PN model are reported for eye comparison (**a**, **b**, **c**, same data as in Fig. 1-a, c, e). Observable MoD reconstructed from the evolutions of G/A MoD under GAN (**d**, **e**,**f**) and GAA (**g**, **h**, **i**) models, and for the three experimental conditions. GAN model perfectly matches the observed MoD. GAA model is to be rejected

Since the three models PN, GAA and GAN can be fitted to correctly predict the evolutions of P/N populations, they can only be discriminated by their capacity to reflect the measured evolutions of observable MoD, namely to account for both MoD and P/N evolutions at the same time. Importantly, we note that the three models do not differ in degrees of freedom, since they all have four parameters (two parameters per tanh function), so differences are only attributable to the difference in their structures. In Fig. 4, we report the reconstruction of observable MoD from the hidden MoD in the GAN and GAA models, along with the MoD directly fitted at the PN level. Visual inspection is sufficient to prefer GAN model over GAA model.

GAN and PN models however differ only slightly. We note a difference at the beginning of the process where nn-divisions rise up later in GAN model than in PN model and seem more adequate. This difference is due to the fact that, in GAN model, nn-divisions are *A*→(*N*,*N*) divisions convoluted by the population of A-cells so they cannot appear before the A-pool has increased. In PN model, they can happen earlier through nn-divisions of P-cells that are present from the beginning.

Overall, if the temporality of the transition among the three modes of division should be controlled by a loss of proliferative capacity in more and more progenitors, then the structure of GAN model should be retained as the best scenario.

### Securing robustness against initial conditions and perturbations

In the calibration of PN model, we have mentioned that the obtained dynamics were highly sensitive to the chosen initial condition (*t*_0_,*P*(*t*_0_)). This is also true for GAN model. In terms of dynamical systems theory, PN and GAN models are *non autonomous linear systems of ODE* because we have considered so far that the evolutions of MoD were decoupled from the evolutions of cells population (MoD were taken as inputs, cells production as outputs) as if MoD were controlled by an external process insensitive to the current amount of cells. In linear models, the final number of produced neurons must be proportional to *P*(*t*_0_), hence the sensitivity.

For the sake of completeness of our modeling proposal, we now speculate about formal refinement that could secure robustness against initial conditions or perturbations. To secure robustness, we have to introduce some feedback control so that the state of the system (the current amount of P/N or G/A/N cells) would directly affect the MoD (see e.g. [13]). For instance, the current amount of *P*-cells could favor the progression to neurogenic divisions, so that the accumulation of *P*-cells by initial proliferation would finally promote more and more nn-divisions. The current amount of *N*-cells could as well favor neurogenic divisions, so that few *N*-cells in the beginning would promote pp-divisions (proliferation) while later accumulation of *N*-cells would progressively dampen proliferation down. We have systematically explored every possible combination [16], and we present here the one that appeared as the most consistent with the data: the one in which the MoD evolutions are controlled by the total amount of cells.

In the terminology of dynamical systems, the PN model with feedback (hereafter denoted PN+fb model) becomes *autonomous non linear*, following :

13 $$ \left\{ \begin{array}{ll} \dot{P}(t) &= (\alpha_{pp}(P,N) - \alpha_{nn}(P,N)) \eta P(t)\\ \dot{N}(t) &= \left(1-(\alpha_{pp}(P,N) - \alpha_{nn}(P,N))\right) \eta P(t) \end{array} \right.  $$

**Fig. 5:**
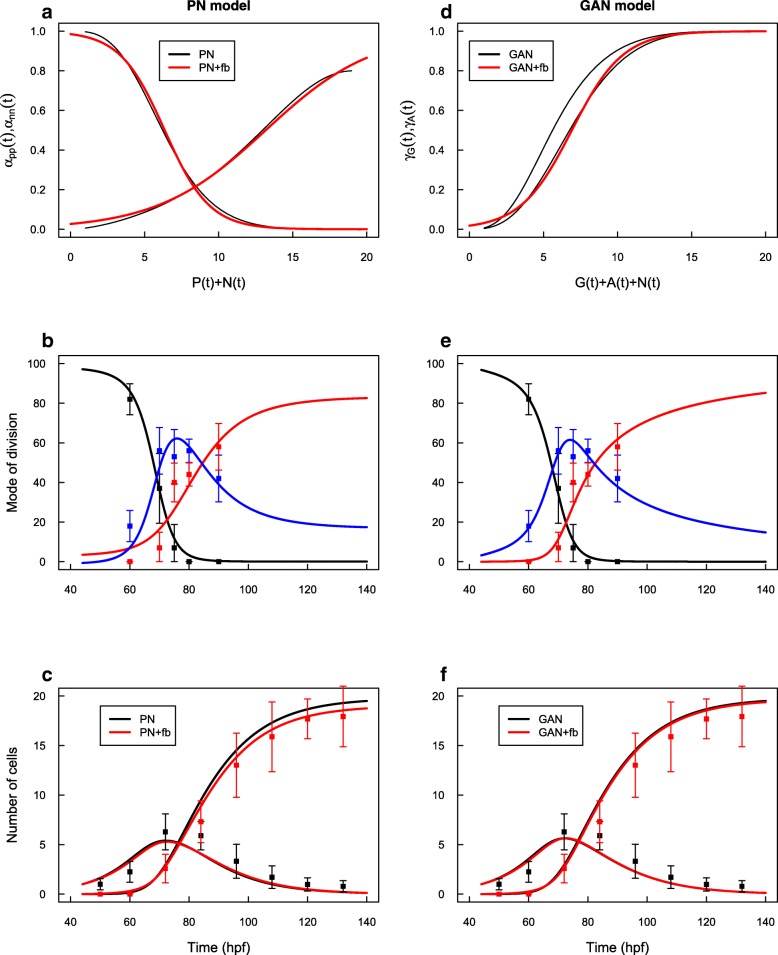
Models PN and GAN with feedback control. Parametric plots of MoD and total amount of cells for PN model (**a**) and GAN model (**d**) (black curves). Feedback control functions with tanh shape were fitted for the two MoD in PN model (red curves), and only one for GAN model (fitting *γ*_*G*_, red curve). The corresponding predictions are given for the evolution of MoD (**b** and **e**), which now result for the dynamics, and the cells population (**c** and **f**). In GAN model, using only one feedback control for the two MoD recovers perfectly the observed data

To establish the form of this feedback control, we plot the MoD as a function of the total amount of cells all along the process in PN model (Fig. 5a, black curves). This suggests, here again, using tanh as an ansatz and the control takes the form:

14 $$ \left\{ \begin{aligned} \alpha_{pp}(P,N) &= \frac{1}{2} \left[1 - \tanh \left(\frac{P+N - \kappa_{pp}}{s_{pp}} \right)\right] \\ \alpha_{nn}(P,N) & = \frac{1}{2} \left[1 + \tanh \left(\frac{P+N - \kappa_{nn}}{s_{nn}} \right)\right] \\ \end{aligned} \right.  $$

The fitted functions are reported in Fig. 5a (red curves), with *κ*_*pp*_=6.4, *κ*_*nn*_=13.2, *s*_*pp*_=3 and *s*_*nn*_=13.2.

Using system (13) with (14), we recover the dynamics of MoD and P/N populations (Fig. 5b and c). Importantly, the MoD are now controlled by the evolution of P/N cells and are not the result of a direct fitting anymore.

Likewise, the GAN model would become:

15 $$ \left\{ \begin{aligned} \dot{G} & = \eta \, \left(1-2\gamma_{G}(G+A+N)\right) G\\ \dot{A} & = \eta \, \left(\gamma_{G}(G+A+N)G -\gamma_{A}(G+A+N)A \right)\\ \dot{N} & = \eta \, \left(\gamma_{G}(G+A+N)G + (1+\gamma_{A}(G+A+N))A \right) \\ \end{aligned} \right.  $$

However, plotting the MoD as a function of the total amount of cells (Fig. 5d, black curves) suggests that both MoD could be driven by one and the same feedback. Denoting *γ*≡*γ*_*G*_(=*γ*_*A*_), GAN+fb is finally:

16 $$ \left\{ \begin{aligned} \dot{G} & = \eta \, G \,\left(1-2\gamma(G+A+N)\right)\\ \dot{A} & = \eta \, (G - A)\, \gamma(G+A+N) \\ \dot{N} & = \eta \, \left[A + (G + A)\,\gamma(G+A+N) \right] \\ \end{aligned} \right.   $$

with

17 $$ \gamma(G,A,N) = \frac{1}{2} \left[1 + \tanh \left(\frac{G+A+N - \kappa_{gan}}{s_{gan}} \right)\right]  $$

The fitted function is reported in Fig. 5d (red curve), with *κ*_*gan*_=6.9 and *s*_*gan*_=3.5. Here again, the MoD are now controlled by the evolution of G/A/N cells and not the result of a direct fitting anymore. Using system (16) with (17), we recover the dynamics of MoD and G/A/N populations (Fig. 5e and f).

By introducing this feedback control, the dynamics would gain robustness against (reasonable) perturbations and converge to the same amount of neurons (three illustrations are given in Additional file 3: Figure S2). In the end, GAN model appears quite relevant as it allows to robustly account for the whole process with only two parameters *κ*_*gan*_,*s*_*gan*_ (in addition to *η*) and it matches the data very well.

## Discussion

Our question was to test whether the progression from proliferation to neurogenic divisions can be explained by a loss of proliferative capacity in an increasing proportion of progenitors. To this end, we have first established a general restriction-free model with progenitors able to perform any kind of division (PN model). Fitting the evolution of its MoDs (PP, PN, NN) from data published by Saade et al. [14], we found smooth MoDs time-profiles that can account for the evolution of the P and N pools reported in [14]. We consider that this general model reflects Sox2 progenitors and HuC/D neurons immunostaining together with the biomarkers which allow differentiating proliferative versus neurogenic divisions [14]. We take it as a benchmark to constrain refined scenarios with heterogeneous progenitors. We note that its general structure is also compatible with a broad description of progenitors / neurons evolution in the neocortex [17, 18]. It should hold as well for other neural tube zones, such as the dorsal area where CDC25B is expressed at the peak of neuronal production [10, 11]. We characterized the behavior of this model under CDC25B GoF experiments carried out by some of us [11] and this gives support to the hypothesis that the action of this phosphatase could be to advance MoD progression, acting there as a maturation factor.

Next we have explored three model structures embedding a loss of proliferative capacity in progenitors, introducing two different progenitors population with the structural constraint that one of them cannot do proliferative divisions. For the three models compatible with this constraint, we have derived the corresponding system of evolution equations. One model (GGA) has been discarded because it could not structurally account for the observed evolutions. For the two other models (GAN, GAA), we have established the correspondence between the evolution of their MoDs and the evolution of the MoDs observed in the benchmark PN model. This correspondence was used to calibrate their parameters and compute their predictions. From these two models, only GAN model appeared to be structurally compatible with observed MoD and P/N evolutions at the same time. In this model, the MoD of *G* and *A* cells evolve at a common pace in the CTL condition, opening the possibility that both are under control of the same regulators. CDC25B GoF accelerates them the same way while CDC25B ^*Δ*CDK^ GoF only delays MoD of *A*-cells.

We note that our modeling proposition displays an important difference with the model proposed by Saade et al. themselves [14] (see also [19]): we do not detect a strong switch of MoD at the population level. Their basic model incorporates an all-or-nothing switch at time *t*^∗^≃80 hpf with only proliferative divisions (pp) before *t*^∗^ and only neurogenic divisions (pn or nn) after *t*^∗^. This is equivalent to a loss of proliferative capacity that would apply to progenitors all at once, at time *t*^∗^. Translated in terms of GAN model, all *G*-cells would instantly become *A*-cells at time *t*^∗^, whatever their phase in cell cycle. They next extend this model to allow smoother transitions, division asynchrony, accelerating cell cycle and a *de novo* incorporation of new progenitors under the induction of Shh. Even with this smoother model, their fitting yields a sharp extinction of pp-divisions at 73 hpf (from 60% to 0% within one hour). It is difficult to determine how this finding is constrained by the initial choice in their basic model, but this predicted evolution of the MoD appears at odd with their experimental observations of MoD and can predict a meaningful evolution of the P/N populations only due to the ad hoc additional source that compensates for the early and sharp extinction of proliferative pp-divisions.

We observe that our model does not incorporate a source of progenitors so the structures of the models are different. We also note that the fitting procedures were not the same. Saade et al. fit the 13 free parameters of their extended model using *an error minimization algorithm with respect to the experimental data* [14] (Extended Experimental Procedures — Mathematical Modeling). As we understand this sentence, they fit the MoD profiles and the source intensity so that the predicted dynamics of the P/N populations matched as close as possible the observed evolution. We have proceeded differently: we have minimized the error between the modeled MoD evolution and the observed MoD evolution, and only then we have checked how the predicted P/N evolutions match or not the observed ones. As a consequence of our procedure, the MoD profiles in the PN and in the GAN models are by construction as close as possible to the observed MoD, and we have no freely adjustable parameters.

Importantly, both procedures have to set an initial condition (i.e. an absolute time 0 at which we fix the initial pool of progenitors), and since proliferative processes are exponential, evolutions of P/N populations are highly sensitive to that choice. We can guess that a small change (by more or less two hours) of that “time 0” in Saade et al. model would have a strong effect upon the required intensity of their additional source. Our first versions of PN and GAN models are to the same extent sensitive to the choice of “time 0”. This sensitivity to initial conditions and timing is due to the fact that these models consider the evolution of MoD as decoupled from the evolution of the populations. The crucial point here is that the relative error for experimental data is the highest at early time, because there are few progenitors then, and the developmental stage is only determinable with an error of the same extent (more or less two hours).

To gain robustness against the indetermination of “time 0”, we have incorporated an hypothetical feedback process so that the evolution of the MoD could be regulated by the state of the system at any time. In these second versions (PN+fb, GAN+fb), there is no need anymore to specify an absolute time scale for the evolution of MoD since it is paced by the evolution of the cells population. This opens new questions about the regulation of CDC25B by upstream signaling, since the maturation factor should itself be under the control of a regulator sensitive to the local amount of cells in the system (e.g. its local extension).

Finally, we advocate that our GAN models (GAN or GAN+fb) indeed incorporate a switch mechanism, but it is specified at the cell level: the switch operates when a daughter of a G-cell loses its proliferative capacity and becomes an A-cell. Considering that a *A*-cell loses its proliferative capacity during the M-phase of its *G*-parent, it would only display its new division modes (AAN, ANN) at its next M-phase. At the population scale, this cell-based MoD switching would then require at least one cell cycle time length to fully display. This is the order of time we observe in the GAN models where the MoD progression at the population level happens over one cell cycle length (12 hours). Under the hypothesis of asynchronous divisions, the smooth progression of MoD in GAN models at the population scale is then compatible with an abrupt signaling event at the cell scale.

From a modeling standpoint (where modeling is used as a way to gain clarity in the face of intricacy [13]), GAN models displays several interesting features compared to PN model. First, CDC25B GoF effect is the same for both models: it hastens MoD progression to neurogenic divisions. Secondly, CDC25B ^*Δ*CDK^ GoF effect can be interpreted straightforwardly in GAN model: the phosphatase unable to interact with its CDK substrate just delays the progression of *A*-cells MoD (it maintains A-cells in self-renewing mode for a longer time). By contrast, CDC25B ^*Δ*CDK^ GoF effect appears as compound in PN model, so it would ask for a convoluted explanation for the differential effect upon advanced pp-divisions and delayed nn-divisions. Thirdly, GAN model can be considered as simpler to interpret from a mechanistic point of view since both types of progenitors display the same monotone evolution of their MoDs. Introducing feedback control to secure some robustness, we showed that MoDs could be under the control of the same signal accumulating monotonously over time, and reflecting directly the system size. With this feedback control, GAN+fb could account for the whole dynamics with only three parameters: *η* which basically represents the unit time of the dynamics, and the two parameters of the feedback control: *κ*_*gan*_ determines the critical size of the total population above which neurogenic divisions become dominant, and *s*_*gan*_ determines how sharp the feedback is. In contrast, PN model would call for a specific explanation of the non monotone evolution of pn-divisions as well as an explanation of the complicated progression among MoDs (five parameters in PN+fb).

Still, the lack of clear discrimination between PN and GAN models is interesting because it shows that the two biological hypotheses (one kind of progenitors able to perform the three kinds of division versus two kinds of progenitors with a loss of proliferative capacity in one kind) can produce predictions compatible with both the MoD and populations evolutions. Since these measures are averages over population, this calls for alternative experimental strategies to support further the plausibility of GAN model. Actually, the two models yield very different predictions if we consider the distributions of content in progenitors/neurons issued from a single initial progenitors (distribution of progenitors/neurons within clones, see Methods “PN and GAN models predictions for clones contents.” section for an illustration). So, the most appealing alternative would be to collect data at the cell scale, either by performing lineage tracing or collecting data about clones contents.

## Methods

### Solving *P*(*t*) from Eq. 3

18 $$ {\begin{aligned} \begin{array}{llll}  P(t) &= P(0)\exp \left[ \eta \int_{0}^{t}(\alpha_{pp}(\tau) - \alpha_{nn}(\tau)) d\tau \right] \\ &= P(0)\exp \left[ \eta \int_{0}^{t} \left(\frac{1}{2} \left[1 - \text{tanh} \left(\frac{\tau - \tau_{pp}}{\sigma_{pp}} \right)\right] \right.\right.\\ & \left. \left.\quad - \frac{1}{2} \alpha_{nn,\infty} \left[1 + \text{tanh} \left(\frac{\tau - \tau_{nn}}{\sigma_{nn}} \right)\right] \right) d\tau \right]\\ &= P(0)\exp \left[ \eta \left(\left(\frac{1}{2} t - \frac{\sigma_{pp}}{2}\ln\left[\frac{\cosh((t-\tau_{pp})/\sigma_{pp})}{\cosh(-\tau_{pp}/\sigma_{pp})}\right] \right)\right.\right.\\ & \left.\left.- \left(\frac{\alpha_{nn,\infty}}{2} t + \frac{\alpha_{nn,\infty}\sigma_{nn}}{2}\ln\left[\frac{\cosh((t-\tau_{nn})/\sigma_{nn})}{\cosh(-\tau_{nn}/\sigma_{nn})} \right] \right) \right) \right]\\ \end{array} \end{aligned}}  $$

hence :

19 $$ {\begin{aligned} \begin{array}{lll}  \frac{P(t)}{P(0)} = \exp\left[{\vphantom{t - \sigma_{pp}\ln\left(\frac{\cosh((t-\tau_{pp})/\sigma_{pp})}{\cosh(-\tau_{pp}/\sigma_{pp})}\right)}}\right. \frac{\eta}{2} &\left({\vphantom{t - \sigma_{pp}\ln\left(\frac{\cosh((t-\tau_{pp})/\sigma_{pp})}{\cosh(-\tau_{pp}/\sigma_{pp})}\right)}}\right. &\left[ t - \sigma_{pp}\ln\left(\frac{\cosh((t-\tau_{pp})/\sigma_{pp})}{\cosh(-\tau_{pp}/\sigma_{pp})}\right) \right]\\ &- \alpha_{nn,\infty} &\left.\left.\left[ t + \sigma_{nn}\ln\left(\frac{\cosh((t-\tau_{nn})/\sigma_{nn})}{\cosh(-\tau_{nn}/\sigma_{nn})}\right) \right] \right) \right] \end{array} \end{aligned}}  $$

### GAN calibration

#### Estimating *γ*_*G*_(*t*) from *α*_*GGG*_(*t*)

Under the *GAN* model, we have at any time the structural correspondence between the two models:

20 $$ \alpha_{GGG}(t)G(t) = \alpha_{pp}(t)P(t)  $$

Setting *G*(0)=1, we have an explicit solution for *G*(*t*) depending on *α*_*GGG*_(*t*) only:

21 $$ G(t) = G(0)\exp\left[\eta \int_{0}^{t} (2\alpha_{GGG}(\tau)-1)d\tau\right]  $$

so we have :

22 $$ \alpha_{GGG}(t)G(0)\exp\left[\eta \int_{0}^{t} (2\alpha_{GGG}(\tau)-1)d\tau\right] = \alpha_{pp}(t)P(t)  $$

We seek a direct expression for *α*_*GGG*_(*t*) despite *α*_*GGG*_(*t*) appears twice, with once in an integral term.

The lhs (left-hand-side) term can be rewritten:

23 $$ \begin{aligned} & \alpha_{GGG}(t)G(0)\exp\left[\eta \int_{0}^{t} (2\alpha_{GGG}(\tau)-1)d\tau\right] \\ = &\alpha_{GGG}(t)G(0)\exp\left[2 \eta \int_{0}^{t} \alpha_{GGG}(\tau)d\tau \right] exp\left[ -\eta t\right] \end{aligned}  $$

Plugging into Eq. 22, and grouping *α*_*GGG*_ terms on the left side, we have:

24 $$ \alpha_{GGG}(t)\exp\!\left[2 \eta \int_{0}^{t} \alpha_{GGG}(\tau)d\tau \!\right] \,=\, \frac{1}{G(0)}\alpha_{pp}(t)P(t)\exp(\eta t)  $$

The lhs can be read as a time-derivative:

25 $$ {\begin{aligned} \frac{d}{dt}\!\left(\frac{1}{2\eta}\exp\!\left[2 \eta \int_{0}^{t} \!\alpha_{GGG}(\tau)d\tau \!\right]\right) \,=\, \frac{1}{G(0)}\alpha_{pp}(t)P(t)\exp(\eta t) \end{aligned}}  $$

Integrating both sides over [0..*t*] :

26 $$ {\begin{aligned} \int_{0}^{t}dt' \frac{d}{dt'}\left(\frac{1}{2\eta}\exp\left[2 \eta \int_{0}^{t'} \alpha_{GGG}(\tau)d\tau \right]\right) = \int_{0}^{t}d\tau\frac{1}{G(0)}\alpha_{pp}(\tau)P(\tau)\exp(\eta \tau) \end{aligned}}  $$

Solving the lhs integral:

27 $$ {\begin{aligned} \frac{1}{2\eta}\exp\left(2 \eta \int_{0}^{t} \alpha_{GGG}(\tau)d\tau \right) -\frac{1}{2\eta} = \int_{0}^{t}d\tau\frac{1}{G(0)}\alpha_{pp}(\tau)P(\tau)\exp(\eta \tau) \end{aligned}}  $$

Rearranging terms and taking the ln of both sides :

28 $$ {\begin{aligned} \int_{0}^{t} \alpha_{GGG}(\tau)d\tau \,=\, \frac{1}{2\eta} \ln\left(1+ \frac{2\eta}{G(0)}\int_{0}^{t}d\tau\alpha_{pp}(\tau)P(\tau)\exp(\eta \tau) \right) \end{aligned}}  $$

Taking the time derivatives of both sides:

29 $$ \begin{aligned} \alpha_{GGG}(t) & = \frac{d}{dt}\left(\frac{1}{2\eta} \ln\left(1+ \frac{2\eta}{G(0)}\int_{0}^{t}d\tau\alpha_{pp}(\tau)P(\tau)\exp(\eta \tau) \right) \right)\\ & = \frac{1}{2\eta} \frac{d}{dt}\ln\left(1+ \frac{2\eta}{G(0)}\int_{0}^{t}d\tau\alpha_{pp}(\tau)P(\tau)\exp(\eta \tau) \right) \end{aligned}  $$

Solving the derivative in the rhs:

30 $$ \alpha_{GGG}(t) = \frac{1}{2\eta} \frac{ \frac{2\eta}{G(0)} \alpha_{pp}(t)P(t)\exp(\eta t) }{1+ \frac{2\eta}{G(0)}\int_{0}^{t}d\tau\alpha_{pp}(\tau)P(\tau)\exp(\eta \tau)}  $$

which simplifies to:

31 $$ \alpha_{GGG}(t) = \frac{ \alpha_{pp}(t)P(t)\exp(\eta t) }{G(0)+ 2\eta\int_{0}^{t}d\tau\alpha_{pp}(\tau)P(\tau)\exp(\eta \tau)}  $$

so we can estimate *γ*_*G*_(*t*)=1−*α*_*GGG*_(*t*) from :

32 $$ {\gamma}_{G}(t) = 1 - \frac{ {\alpha}_{pp}(t){P}(t)\exp(\eta t) }{G(0)+ 2\eta\int_{0}^{t}d\tau{\alpha}_{pp}(\tau){P}(\tau)\exp(\eta \tau)}  $$

using the evolution of *α*_*pp*_(*t*) (Eq. 4) and *P*(*t*) (Eq. 6) obtained in the three experimental conditions.

The results are given in Additional file 4: Figure S3 (green curves).

We note that calibrating *γ*_*G*_(*t*) by this method only yields a raw unparameterized temporal series. The obtained results however strongly suggest an hyperbolic tangent shape (tanh) as an ansatz for this evolution, following:

33 $$ \gamma'_{G}(t, \tau_{G}, \sigma_{G}) = \frac{1}{2} \left[1 + \tanh \left(\frac{t - \tau_{G}}{\sigma_{G}} \right)\right]  $$

To parametrize *γG*′, we seek the pair $(\tau ^{*}_{G}, \sigma ^{*}_{G})$ that minimizes the error between the evolution predicted by system (7) and the observed evolutions in the PN model (1). Using Eq. 22, we then seek to minimize the error function:

34 $$ {\begin{aligned} E(\tau_{G},\sigma_{G})& = \int_{0}^{T}dt \left({\vphantom{\int_{0}^{t}}} \hat{\alpha}_{pp}(t)\hat{P}(t) \right. \\ &\qquad \left. - \left(1-\gamma^{\prime}_{G}(t, \tau_{G}, \sigma_{G})\right)G(0)\exp\left[\eta \int_{0}^{t} (1-2\gamma^{\prime}_{G}(\tau, \tau_{G}, \sigma_{G}))d\tau\right] \right)^{2} \end{aligned}}  $$

We used Nelder-Mead optimization from R-software ’optim’ using time-discretized series with *Δt*=0.01 hour, *T*=96h.

The tanh ansatz appears to match perfectly the analytically derived time series (Additional file 4: Figure S3, black curves), so we parametrize *γ*_*G*_ with the corresponding parameters $\tau ^{*}_{G}$ and $\sigma ^{*}_{G}$:

35 $$ \gamma_{G}(t) = \frac{1}{2} \left[1 + \tanh \left(\frac{t - \tau^{*}_{G}}{\sigma^{*}_{G}} \right)\right]  $$

The fitted values for $\left (\tau ^{*}_{G}, \sigma ^{*}_{G}\right)$ under the three experimental conditions are given in Table 2.

#### Estimating *γ*_*A*_

Considering the parameter for the evolution of population *A*, the structural correspondence is:

36 $$ \begin{aligned} \gamma_{A}(t) \, \frac{A(t)}{G(t) + A(t)} = \alpha_{nn}(t) \end{aligned}  $$

Here, *A*(*t*) is governed by:

37 $$ \begin{aligned} \dot{A}(t) &= \eta \, \left[ \gamma_{G}(t) G(t) - \gamma_{A}(t) A(t)\right] \end{aligned}  $$

so we can not obtain an explicit solution for *A*(*t*) as a function of *γ*_*A*_(*t*).

Hence, we proceed with the ansatz method, testing a tanh shape for *γ*_*A*_(*t*), following:

38 $$ \gamma_{A} (t,\tau_{A},\sigma_{A}) = \frac{1}{2} \left[1 + \text{tanh} \left(\frac{t - \tau_{A}}{\sigma_{A}} \right)\right]  $$

Since *γ*_*G*_(*t*) and *G*(*t*) are known from section above, we can then use Eq. 37 to numerically solve the evolution of population A, once given *γ*_*A*_(*t*,*τ*_*A*_,*σ*_*A*_). We denote *A*(*t*,*τ*_*A*_,*σ*_*A*_) this numerical solution.

We then seek the pair $(\tau ^{*}_{A},\sigma ^{*}_{A})$ that minimizes the square error:

39 $$ {\begin{aligned} E(\tau_{A},\sigma_{A}) \,=\, \int_{0}^{T}dt \ \left({\alpha}_{nn}(t) - \gamma_{A}(t,\tau_{A},\sigma_{A})\frac{A(t,\tau_{A},\sigma_{A})}{G(t) + A(t,\tau_{A},\sigma_{A})} \right)^{2} \end{aligned}}  $$

using Nelder-Mead optimization over time-discretized series (with *Δt*=0.01 hour, *T*=96h).

The fitted values for $(\tau ^{*}_{A}, \sigma ^{*}_{A})$ under the three experimental conditions are given in Table 2.

The tanh ansatz seems to be highly relevant since the predicted evolutions for the evolution of the P,N populations are well in accordance with the observed ones (Fig. 2).

### GAA calibration

#### Estimating *γ*_*G*_(*t*) from *α*_*GGG*_(*t*)

Under GAA model, we have the structural correspondence between the two models:

40 $$ \begin{aligned} \alpha_{pp}(t) & = (1-\gamma_{G}(t)) \, \frac{G(t)}{G(t) + A(t)} + \gamma_{G}(t)\, \frac{G(t)}{G(t) + A(t)}\\ & = \frac{G(t)}{G(t) + A(t)} \end{aligned}  $$

Using *P*(*t*)=*G*(*t*)+*A*(*t*), we obtain:

41 $$ \begin{aligned} G(t) & = \alpha_{pp}(t) \left(G(t) + A(t) \right) = \alpha_{pp}(t) P(t) \end{aligned}  $$

Setting *G*(0)=1, we have an explicit solution for *G*(*t*) depending on *γ*_*G*_(*t*) only:

42 $$ \begin{aligned} G(t) = G(0)\exp\left[ \eta\int_{0}^{t}(2\alpha_{GGG}(\tau)-1) d\tau \right] \end{aligned}  $$

so we have :

43 $$ G(0)\exp\left[\eta \int_{0}^{t} (2\alpha_{GGG}(\tau)-1)d\tau\right] = \alpha_{pp}(t)P(t)  $$

We seek a direct expression for *α*_*GGG*_(*t*).

From Eq. 43, we have:

44 $$ \int_{0}^{t} \alpha_{GGG}(\tau)d\tau = \frac{1}{2\eta}\ln\left[ \frac{1}{G(0)} \alpha_{pp}(t)P(t)\exp(\eta t) \right]  $$

Taking the time derivatives of both sides, we obtain:

45 $$ \alpha_{GGG}(t) = \frac{1}{2\eta}\left[ \frac{\dot{\alpha}_{pp}(t)P(t)+\alpha_{pp}(t)\dot{P}(t)}{\alpha_{pp}(t)P(t)} + \eta \right]  $$

so we can estimate *γ*_*G*_(*t*)=1−*α*_*GGG*_(*t*) from this expression, using the evolution of *α*_*pp*_(*t*) (Eq. 4) and *P*(*t*) (Eq. 6) obtained in the three experimental conditions, and numerical derivation for $\dot {\alpha }_{pp}(t)$ and $\dot {P}(t)$.

The results are given in Additional file 5: Figure S4 (green curves). It appeared that the estimated functions *γ*_*G*_(*t*) violate the constraint of belonging to the interval [0..1]. This is the sign that this model can not at the same time be adjusted to the MoD of PN model and predict correct evolutions for the P,N populations.

Notwithstanding, we proceeded with the ansatz method in order to examine which *γ*_*G*_(*t*) would yield correct predictions for the P,N populations. Setting a tanh shape for it,

46 $$ \gamma_{G}(t, \tau_{G}, \sigma_{G}) = \frac{1}{2} \left[1 + \text{tanh} \left(\frac{t - \tau_{G}}{\sigma_{G}} \right)\right]  $$

we then seek the pair $\left (\tau ^{*}_{G},\sigma ^{*}_{G}\right)$ that minimizes the error of prediction upon $\hat {\alpha }_{pp}(t) \hat {P}(t)$, given by:

47 $$ {\begin{aligned} E(\tau_{G},\sigma_{G}) = \int_{0}^{T}dt \left(\hat{\alpha}_{pp}(t)\hat{P}(t) - \exp \left[\int_{0}^{t}d\tau \ \eta \left(1-2 \gamma_{G}(\tau, \tau_{G}, \sigma_{G}) \right) \right] \right)^{2} \end{aligned}}  $$

We used Nelder-Mead optimization over time-discretized series (with *Δt*=0.01 hour, *T*=96h).

The fitted values for $\left (\tau ^{*}_{G}, \sigma ^{*}_{G}\right)$ under the three experimental conditions are given in Table 3.

#### Estimating *γ*_*A*_

To estimate *γ*_*A*_ for model GAA, we proceeded the same way as for the model GAN, except that we used:

48 $$ \begin{aligned} \dot{A}(t) &= \eta \, \left[ 2 \gamma_{G}(t) G(t) - \gamma_{A}(t) A(t)\right] \end{aligned}  $$

The fitted values for $\left (\tau ^{*}_{A}, \sigma ^{*}_{A}\right)$ under the three experimental conditions are given in Table 3.

The tanh ansatz can then be adjusted to produce predicted evolutions of P,N populations in accordance with the observed ones (Fig. 3).

### PN and GAN models predictions for clones contents.

We illustrate here that even if PN and GAN models yield the same predictions regarding the averaged populations of progenitors and neurons they produce, they however differ in predictions if we consider the distributions of contents in progenitors/neurons issued from a single initial progenitors (distribution of progenitors/neurons within clones). Due to the stochastic nature of the MoD embedded in the model, each initial progenitor should indeed produce a stochastic tree of descent. Clone contents are then defined here by the pairs (number of progenitors, number of neurons) obtained after a number *C* of cell cycles. For instance, if an initial *P*-cell undergoes a first division of PN MoD, it will produce one neuron of generation 1, and one progenitor of generation 1. If the latter undergoes a nn-division, it will produce two neurons of generation 2, so in the end the content of the clone after two cell cycles will be (0,3). Another initial *P*-cell could undergo a first pp-division producing two progenitors of generation 1; if one of them undergoes a pp-division, and the other one undergoes a nn-division, this will end in a (2,2) clone content at generation 2. We can then compute the statistical distribution of these contents by repeatedly sampling the stochastic production of trees of descent.

To build an illustration of the above process in a simple manner, we consider here MoD that are fixed in time, and we compute the distribution of clones contents produced by *G*-cells after two cell cycles (we used 10^6^ stochastic samples under each model). To make predictions comparable, we fix the MoD under both models including feedback control at the values they have at the same time point. We chose that time point as 68 hpf, i.e. the time at which MoD of *G*-cells becomes predominantly neurogenic in GAN model (the conclusions are independent of that choice). Hence, the MoD values we used are: *α*_*pp*_=0.4975 and *α*_*nn*_=0.135 in PN model, and *γ*_*G*_=*γ*_*A*_=0.446 in GAN model. Importantly for the comparison, both models expectedly predict similar amounts of averaged number of *P*/*N*-cells after two generations (*P*_2_=1.86 and *N*_2_=1.51 with PN model, and *P*_2_=1.96 and *N*_2_=1.59 with GAN model; in both cases, the observed proportions of progenitors are 55.2% at generation 2).

The expected clone contents are reported in Table 4 for PN model and in Table 5 for GAN model (in these tables, empty cells are unreachable contents). The different clone contents would not appear with same probabilities under the two scenarios. For instance, clones made of (*P*,*N*)=(0,3) should appear in 5% of clones under PN model whereas they should appear in 20% of clones under GAN model. Even more discriminative, the content (*P*,*N*)=(2,1) which is the most expected under PN model should not appear at all under GAN model (at generation 2 of a *G*-cell). Further theoretical work is needed to build completely usable predictions to be compared with experimental data, taking into account asynchronous divisions, time mixing of G/A populations in the GAN model and MoD evolving with time or by feedback control.

**Table 4:** PN predictions for the expected fraction of each clone contents (*P*,*N*) at generation 2

	N				
P	0	1	2	3	4
0			13.4	04.9	00.9
1			13.4	04.9	
2		18.3	13.4		
3		18.2			
4	12.3

**Table 5:** GAN predictions for clone contents at generation 2

	N				
P	0	1	2	3	4
0				19.9	
1			24.8		
2			11.0		
3		27.3			
4	17.0				

## Additional files

Additional file 1 All data and codes used to generate the figures are contained in the R script DataAndCode.R. (R 72 kb)

Additional file 2 Simplified GAN Model. Same legend as Fig. 2. The simplified version of GAN model is when a *A*-cell only performs *A*→(*N*,*N*) divisions, so *γ*_*A*_(*t*) is forced to the value 1 at any time. This simplified version yields predictions which are practically identical to GAN predictions, except a slight difference in the early rise of nn-divisions, and an incorrect prediction for the MoD under the GoF of mutated CDC25B experiment (i). (PDF 22 kb)

Additional file 3 In all cases, the models with feedback control converge to about the same final amount of neurons. (PDF 21 kb)

Additional file 4 Analytical and least-square fitted *γ*_*G*_(*t*)for GAN model. Predicted evolution of *γ*_*G*_(*t*)obtained by analytical inversion are reported in green. Fitted tanh ansatz are reported in black and perfectly overlap. (PDF 199 kb)

Additional file 5 Analytical and least-square fitted *γ*_*G*_(*t*) for GAA model. Same conventions as in Additional file 4: Figure S3. In GAA model, the analytical inversion of *γ*_*G*_(*t*)yields an evolution that violates the constraint of belonging to the interval [0..1] (green curves). Fitted tanh ansatz are reported in black. (PDF 187 kb)

## Abbreviations

BMP: Bone morphogenetic protein
CDC25B: Cell division cycle 25 B phosphatase
CDK: Cyclin-dependent kinases
CNS: Central nervous system
CTL: Control
MoD: Mode of division
NSC: Neural stem cells
Shh: Sonic hedgehog

## References

[1] Doe CQ. Temporal Patterning in the Drosophila CNS. Annu Rev Cell Dev Biol. 2017; 33:219–40. 10.1146/annurev-cellbio-111315-125210. 10.1146/annurev-cellbio-111315-125210 28992439

[2] Kang KH, Reichert H. Control of neural stem cell self-renewal and differentiation in Drosophila. Cell Tissue Res. 2015; 359(1):33–45. 10.1007/s00441-014-1914-9. 10.1007/s00441-014-1914-9 24902665

[3] Syed MH, Mark B, Doe CQ. Playing Well with Others: Extrinsic Cues Regulate Neural Progenitor Temporal Identity to Generate Neuronal Diversity. Trends Genet. 2017; 33(12):933–42. 10.1016/j.tig.2017.08.005. 10.1016/j.tig.2017.08.005 PMC5701851 28899597

[4] Molyneaux BJ, Arlotta P, Menezes JRL, Macklis JD. Neuronal subtype specification in the cerebral cortex,. Nat Rev Neurosci. 2007; 8(6):427–37. 10.1038/nrn2151. 10.1038/nrn2151 17514196

[5] Taverna E, Götz M, Huttner WB, Vol. 30. The Cell Biology of Neurogenesis: Toward an Understanding of the Development and Evolution of the Neocortex; 2014. pp. 465–502. 10.1146/annurev-cellbio-101011-155801. http://www.annualreviews.org/doi/10.1146/annurev-cellbio-101011-155801. 10.1146/annurev-cellbio-101011-155801 25000993

[6] Kicheva A, Bollenbach T, Ribeiro A, Valle HP, Lovell-Badge R, Episkopou V, Briscoe J. Coordination of progenitor specification and growth in mouse and chick spinal cord. Science. 2014; 345(6204):1254927. 10.1126/science.1254927. 10.1126/science.1254927 PMC4228193 25258086

[7] Kicheva A, Briscoe J. Developmental Pattern Formation in Phases. Trends Cell Biol. 2015; 25(10):579–91. 10.1016/j.tcb.2015.07.006. 10.1016/j.tcb.2015.07.006 26410404

[8] Zagorski M, Tabata Y, Brandenberg N, Lutolf MP, Tkačik G, Bollenbach T, Briscoe J, Kicheva A. Decoding of position in the developing neural tube from antiparallel morphogen gradients. Science. 2017; 356(6345):1379–83. 10.1126/science.aam5887. 10.1126/science.aam5887 PMC5568706 28663499

[9] Agius E, Bel-Vialar S, Bonnet F, Pituello F. Cell cycle and cell fate in the developing nervous system: the role of CDC25B phosphatase. Cell Tissue Res. 2015; 359(1):201–13. 10.1007/s00441-014-1998-2. 10.1007/s00441-014-1998-2 25260908

[10] Peco E, Escude T, Agius E, Sabado V, Medevielle F, Ducommun B, Pituello F. The CDC25B phosphatase shortens the G2 phase of neural progenitors and promotes efficient neuron production. Development. 2012; 139(6):1095–104. 10.1242/dev.068569. 10.1242/dev.068569 22318230

[11] Bonnet F, Molina A, Roussat M, Azais M, Vialar S, Gautrais J, Pituello F, Agius E. Neurogenic decisions require a cell cycle independent function of the CDC25B phosphatase. eLife. 2018; 7. 10.7554/eLife.32937. 10.7554/eLife.32937 PMC6051746 29969095

[12] Danesin C, Soula C. Moving the Shh Source over Time: What Impact on Neural Cell Diversification in the Developing Spinal Cord?J Dev Biol. 2017; 5(2):4. 10.3390/jdb5020004. 10.3390/jdb5020004 PMC5831764 29615562

[13] Lander AD, Gokoffski KK, Wan FYM, Nie Q, Calof AL. Cell lineages and the logic of proliferative control. PLoS Biol. 2009; 7(1):15. 10.1371/journal.pbio.1000015. 10.1371/journal.pbio.1000015 PMC2628408 19166268

[14] Saade M, Gutiérrez-Vallejo I, LeDréau G, Rabadán MA, Miguez DG, Buceta J, Martí E. Sonic hedgehog signaling switches the mode of division in the developing nervous system. Cell Rep. 2013; 4(3):492–503. 10.1016/j.celrep.2013.06.038. 10.1016/j.celrep.2013.06.038 23891002

[15] Molina A, Pituello F. Playing with the cell cycle to build the spinal cord. Dev Biol. 2016; 432(1):14–23. 10.1016/j.ydbio.2016.12.022. 10.1016/j.ydbio.2016.12.022 28034699

[16] Azaïs M. Embryogenèse de la moelle épinière: de la dynamique collective observable à une proposition de modèle comportemental à l’échelle cellulaire. Phd thesis (in french), Université de Toulouse. 2018. 10.13140/RG.2.2.25828.83848.

[17] Takahashi T, Nowakowski RS, Caviness VS. The leaving or Q fraction of the murine cerebral proliferative epithelium: a general model of neocortical neuronogenesis,. J Neurosci. 1996; 16(19):6183–96. 10.1523/JNEUROSCI.16-19-06183.1996. 10.1523/JNEUROSCI.16-19-06183.1996 PMC6579174 8815900

[18] Nowakowski RS, Caviness VS, Takahashi T, Hayes NL. Population dynamics during cell proliferation and neuronogenesis in the developing murine neocortex. Results Probl Cell Differ. 2002; 39:1–25. 10.1007/978-3-540-46006-0_1. 10.1007/978-3-540-46006-0_1 12353465

[19] Míguez DG. A Branching Process to Characterize the Dynamics of Stem Cell Differentiation. Sci Rep. 2015; 5(1):13265. 10.1038/srep13265. 10.1038/srep13265 PMC4541069 26286123

