# Supplementary figures and images for: Timing the spinal cord development with neural progenitor cells losing their proliferative capacity: a theoretical analysis
Source: Neural Dev. 2019 Mar 13;14:7. doi: 10.1186/s13064-019-0131-3 (PMC6417072; doi:10.1186/s13064-019-0131-3)

**a – CTL**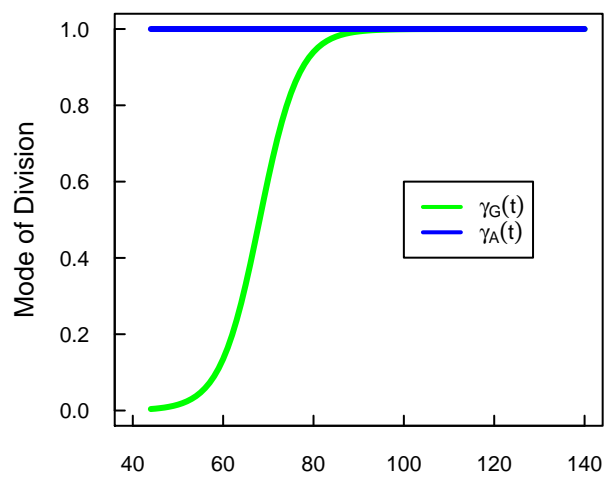**b**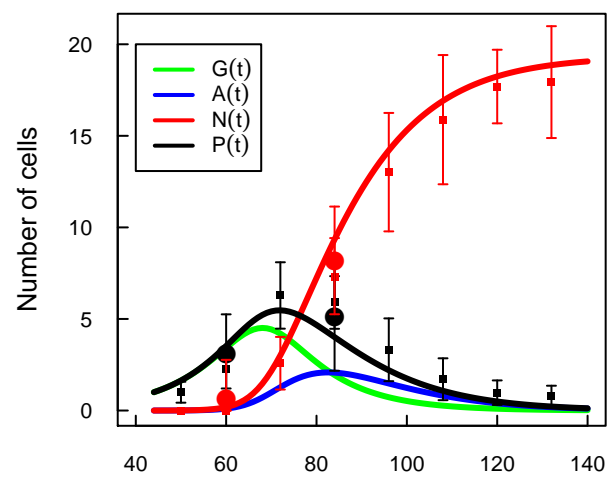**c**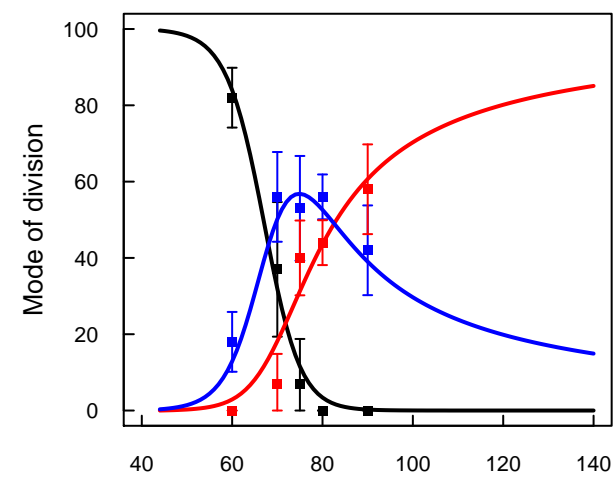**d – GoF**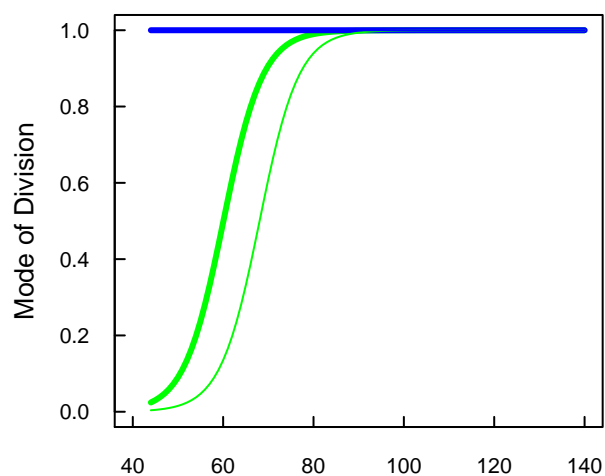**e**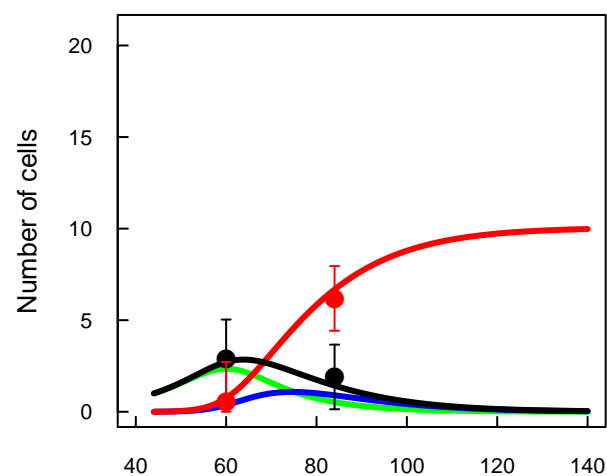**f**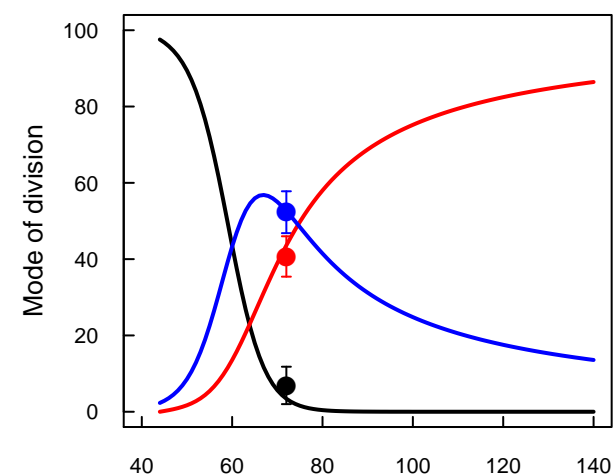**g – GoF- $\Delta$ CDK**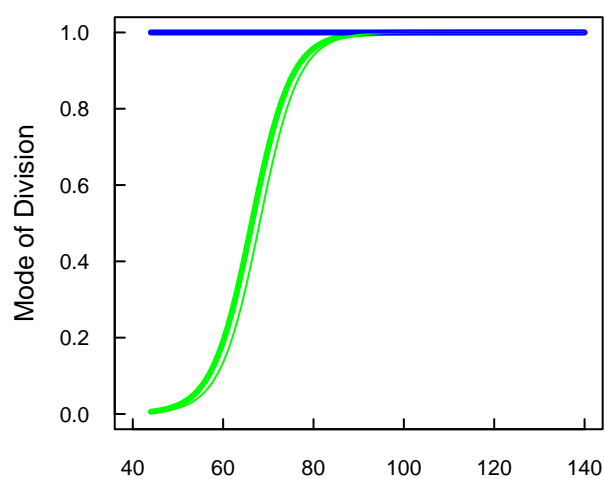**h**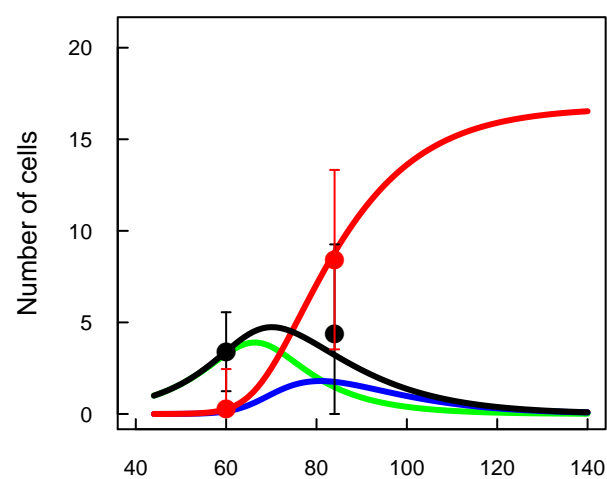**i**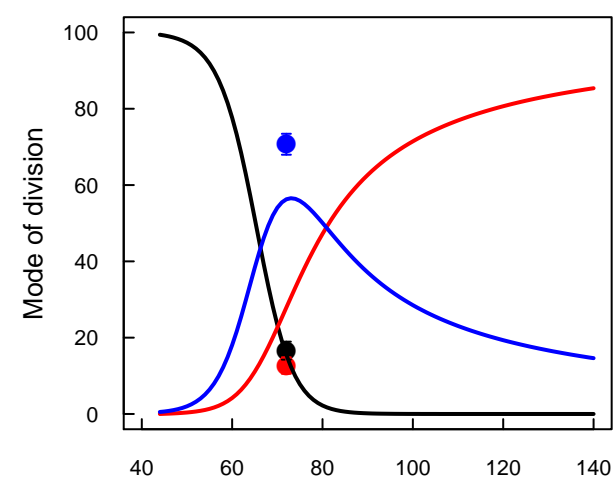

Supplement: Supplementary file 2 — Simplified GAN Model. Same legend as Fig. 2. The simplified version of GAN model is when a A-cell only performs A→(N,N) divisions, so γA(t) is forced to the value 1 at any time. This simplified version yields predictions which are practically identical to GAN predictions, except a slight difference in the early rise of nn-divisions, and an incorrect prediction for the MoD under the GoF of mutated CDC25B experiment (i). (PDF 22 kb) [file 13064_2019_131_MOESM2_ESM.pdf]

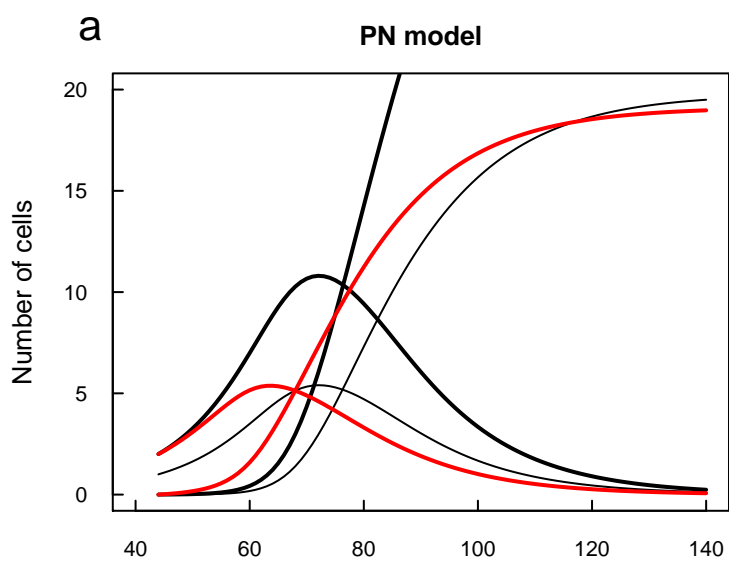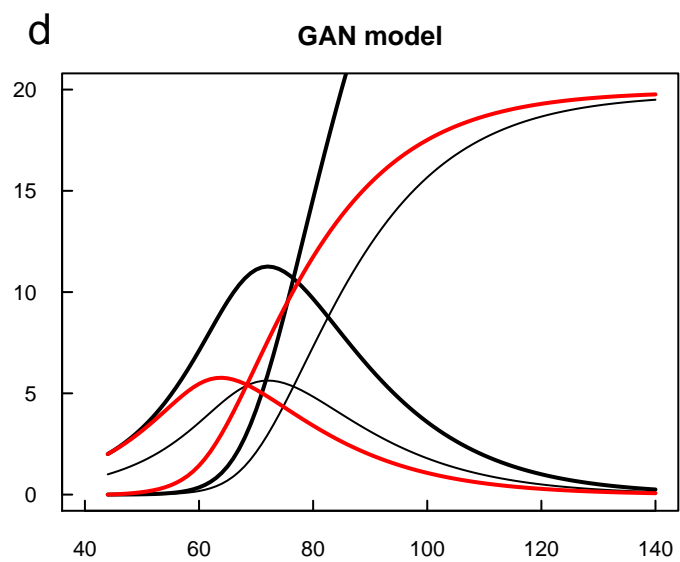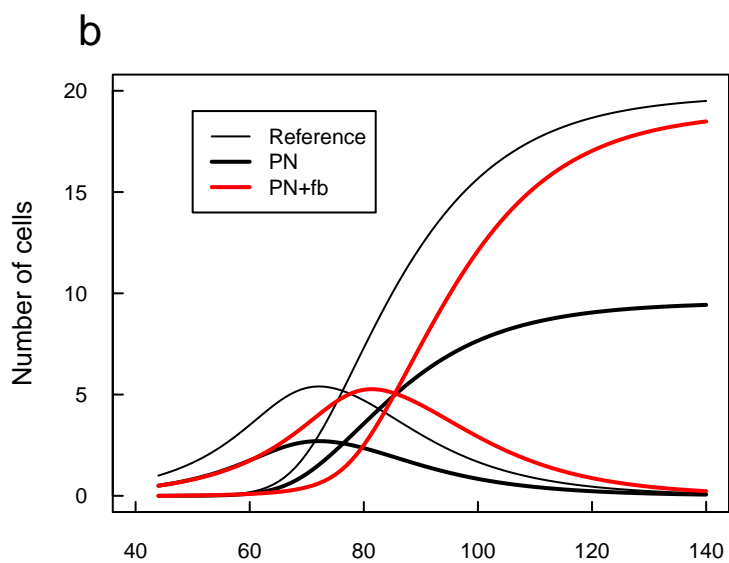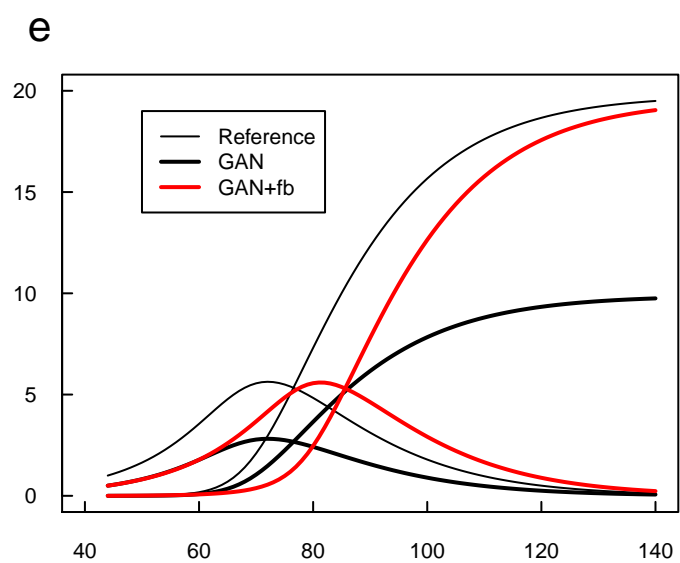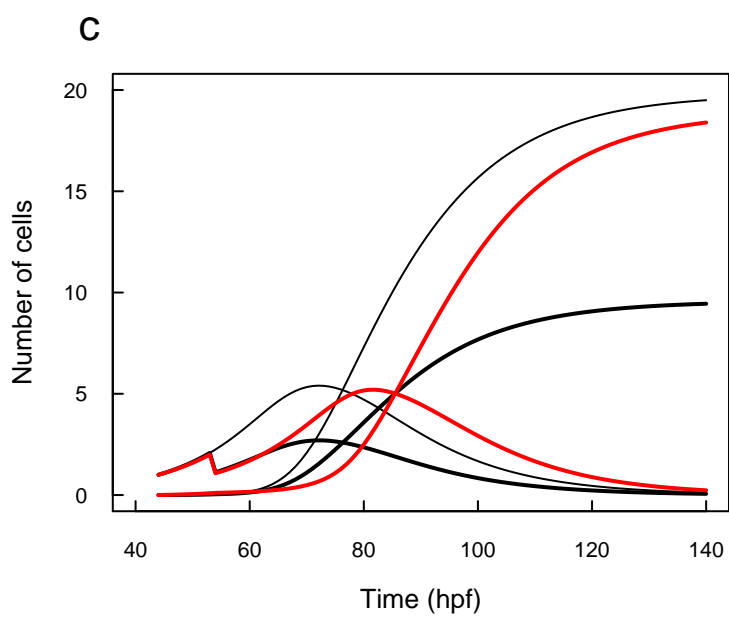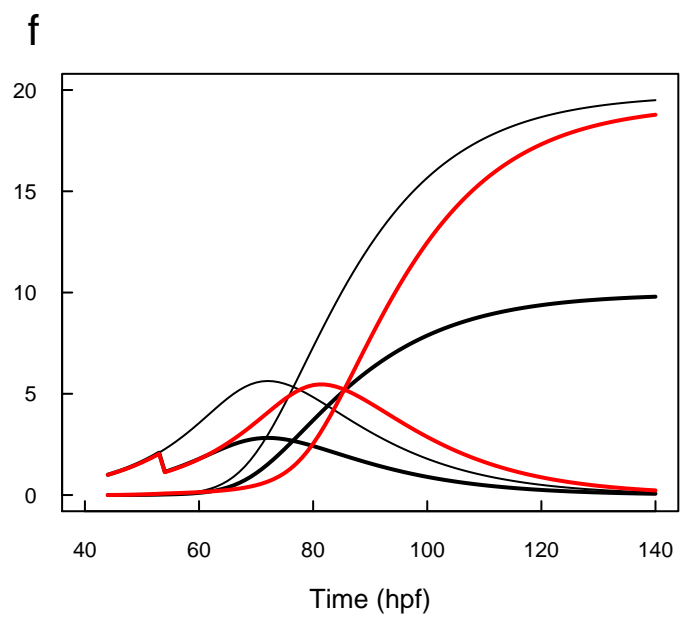

Supplement: Supplementary file 3 — In all cases, the models with feedback control converge to about the same final amount of neurons. (PDF 21 kb) [file 13064_2019_131_MOESM3_ESM.pdf]

CTL

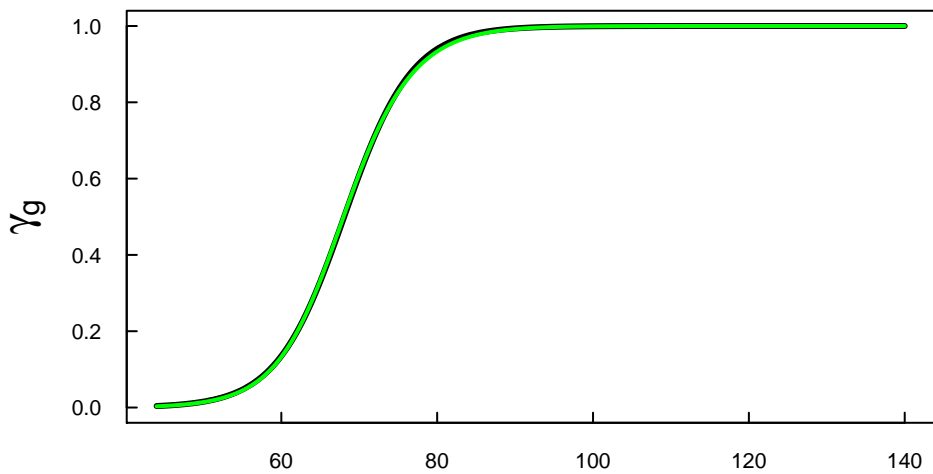

GoF

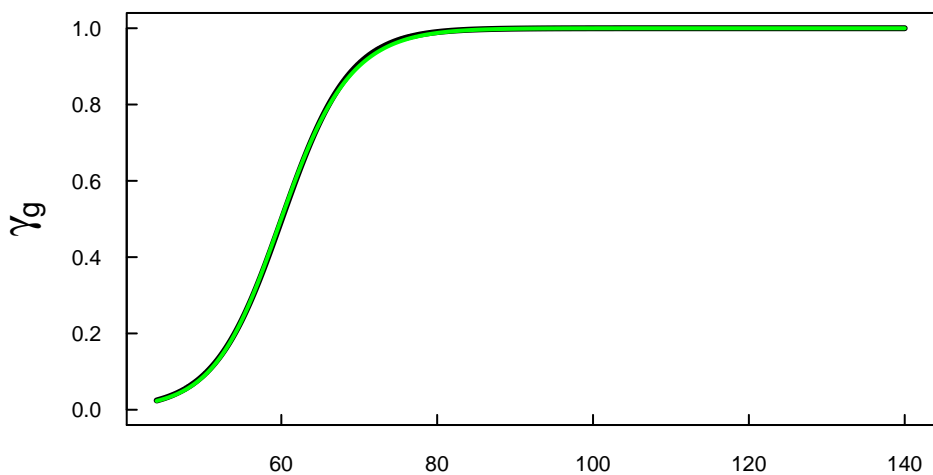GoF- $\Delta$ CDK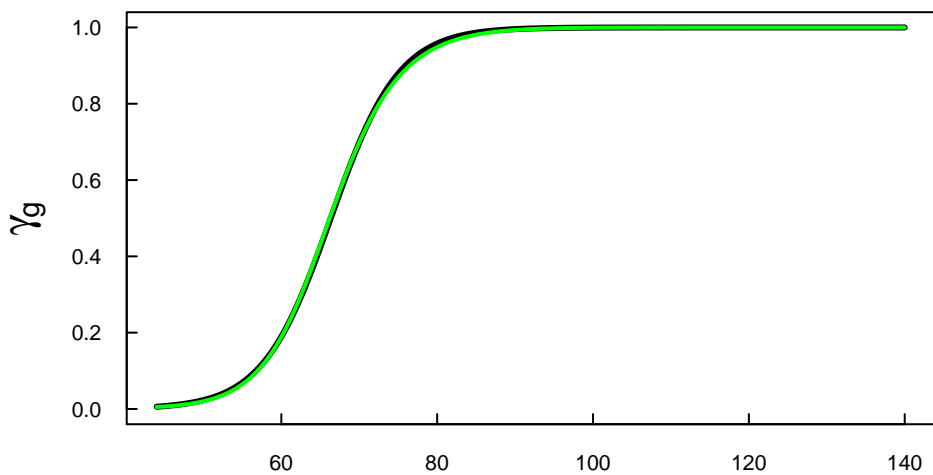

Time (hpf)

Supplement: Supplementary file 4 — Analytical and least-square fitted γG(t)for GAN model. Predicted evolution of γG(t)obtained by analytical inversion are reported in green. Fitted tanh ansatz are reported in black and perfectly overlap. (PDF 199 kb) [file 13064_2019_131_MOESM4_ESM.pdf]

CTL

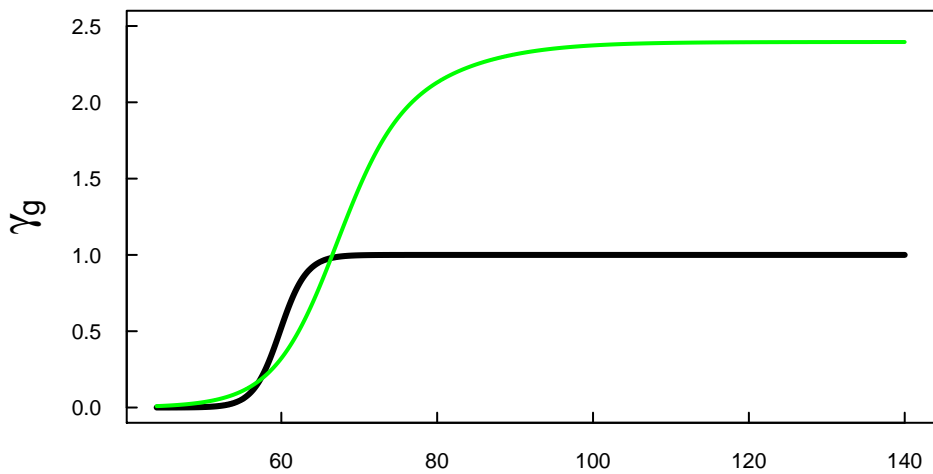

GoF

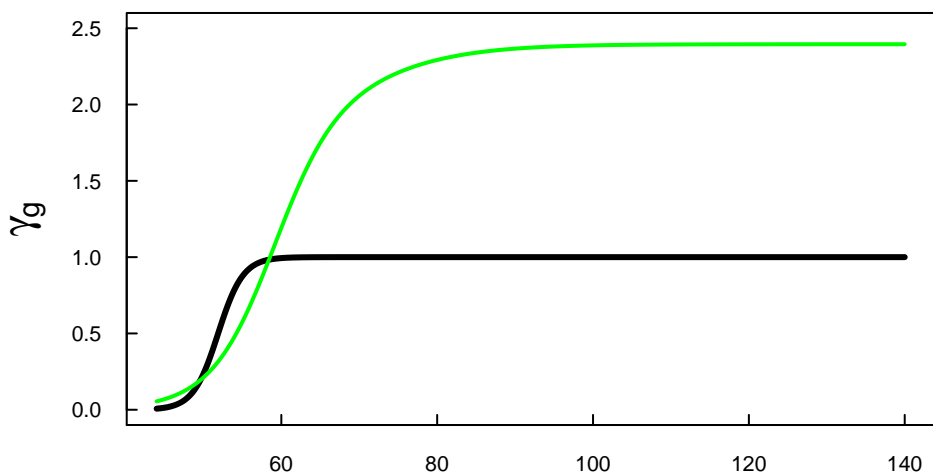GoF- $\Delta$ CDK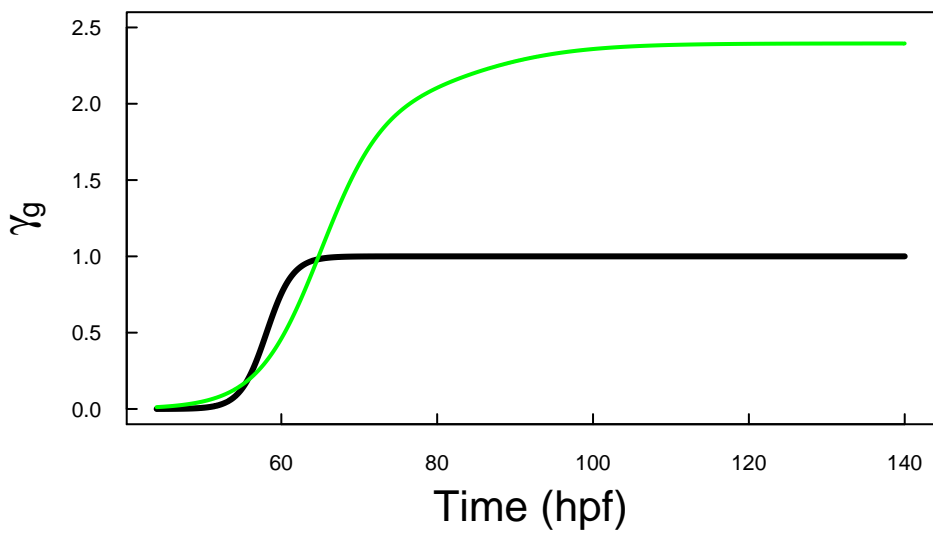

Supplement: Supplementary file 5 — Analytical and least-square fitted γG(t) for GAA model. Same conventions as in Additional file 4: Figure S3. In GAA model, the analytical inversion of γG(t)yields an evolution that violates the constraint of belonging to the interval [0..1] (green curves). Fitted tanh ansatz are reported in black. (PDF 187 kb) [file 13064_2019_131_MOESM5_ESM.pdf]
